# Computational study of the binding orientation and affinity of noncovalent inhibitors of the papain-like protease (PLpro) from SARS-CoV-1 considering the protein flexibility by using molecular dynamics and cross-docking

**DOI:** 10.3389/fmolb.2023.1215499

**Published:** 2023-06-23

**Authors:** Luis Castillo-Campos, José Luis Velázquez-Libera, Julio Caballero

**Affiliations:** Centro de Bioinformática, Simulación y Modelado (CBSM), Facultad de Ingeniería, Universidad de Talca, Talca, Chile

**Keywords:** papain-like protease, PLpro inhibitors, SARS-CoV, naphthalene-derived compounds, docking, molecular dynamics

## Abstract

The papain-like protease (PLpro) from zoonotic coronaviruses (CoVs) has been identified as a target with an essential role in viral respiratory diseases caused by Severe Acute Respiratory Syndrome-associated coronaviruses (SARS-CoVs). The design of PLpro inhibitors has been proposed as an alternative to developing potential drugs against this disease. In this work, 67 naphthalene-derived compounds as noncovalent PLpro inhibitors were studied using molecular modeling methods. Structural characteristics of the bioactive conformations of these inhibitors and their interactions at the SARS-CoV-1 PLpro binding site were reported here in detail, taking into account the flexibility of the protein residues. Firstly, a molecular docking protocol was used to obtain the orientations of the inhibitors. After this, the orientations were compared, and the recurrent interactions between the PLpro residues and ligand chemical groups were described (with LigRMSD and interaction fingerprints methods). In addition, efforts were made to find correlations between docking energy values and experimentally determined binding affinities. For this, the PLpro was sampled by using Gaussian Accelerated Molecular Dynamics (GaMD), generating multiple conformations of the binding site. Diverse protein conformations were selected and a cross-docking experiment was performed, yielding models of the 67 naphthalene-derived compounds adopting different binding modes. Representative complexes for each ligand were selected to obtain the highest correlation between docking energies and activities. A good correlation (*R* ^2^ = 0.948) was found when this flexible docking protocol was performed.

## 1 Introduction

Zoonotic coronaviruses (CoVs) are important viral pathogens whose most recent species, the Severe Acute Respiratory Syndrome (SARS)-CoV-2, has been causing a worldwide emergency due to its rapid spread since the end of 2019. Previous CoV events caused by the SARS-CoV-1 (2002–2003) and the Middle East Respiratory Syndrome (MERS)-CoV (2012) were antecedents that showed the danger constituted by CoVs. After the SARS-CoV-1 appeared in Guangdong province in China in November 2002, affecting three continents and causing many deaths (Wang and Chang, 2004), researchers investigated the mechanisms of viral infection to discover options to provide treatment for patients infected with zoonotic CoVs. The results of these investigations made it possible to identify molecular targets currently being investigated to find specific drugs against CoVs. Research to modulate these targets has included the repurposing of already approved drugs (De Savi et al., 2020; Gordon et al., 2020; Indari et al., 2022; Khataniar et al., 2022) and the design of new specific drugs (Cannalire et al., 2022).

Infection with CoVs triggers the encoding of several protein targets with recognized functions relevant to the virus infection. The proteases 3CLpro and PLpro were identified as responsible for preprocessing translated multidomain polyproteins from the viral RNA genome (Hilgenfeld, 2014; Zhu et al., 2021). Since 2003, details of the structure and functions of 3CLpro have been reported; its structural and mechanistic aspects have been elucidated, offering multiple avenues as starting points for the design of antiviral compounds directed against CoVs (Ullrich and Nitsche, 2020). On the other hand, the less studied PLpro also plays critical biochemical events for coronavirus replication. It is vital in viral pathogenesis and is associated with processes of deubiquitination and deISGylation of host cell proteins (Báez-Santos et al., 2015). In association with viral protein processing, its enzymatic activity triggers the host antiviral immune response antagonism.

The architecture of PLpro consists of four domains: the palm domain, the thumb, the fingers, and an independent terminal domain similar to the ubiquitin domains. The binding site of PLpro is at the intersection between the palm and thumb domains (Ratia et al., 2006), formed by a catalytic triad composed of the residues Cys112-His273-Asp287 (in the SARS-CoV-1 PLpro) and subsites that can be specifically occupied by the substrate RLRGG (the C-terminus of ubiquitin). Closed and open conformations of the binding site are available because of structural changes in the six-residue BL2 loop, modulating substrate recognition (Chaudhuri et al., 2011).

Targeting PLpro has become an attractive strategy to stop the viral replication and infection caused by CoVs. In this sense, the design of PLpro inhibitors has been proposed (Calleja et al., 2022). In recent years, Ratia et al. synthesized a series of noncovalent naphthalene-derived compounds as SARS-CoV-1 PLpro inhibitors by high-throughput screening (Ratia et al., 2008; Ghosh et al., 2009; Ghosh et al., 2010; Báez-Santos et al., 2014a). They act as reversible competitive PLpro inhibitors by binding to the S_3_-S_4_ subsites (Supplementary Figure S1). When bound, these compounds induce the reorientation of the Y269 side chain, generating the closure of the BL2 loop. Some of these compounds were co-crystallized with PLpro, allowing an initial source to generate more structural information explaining what structural aspects contribute to the differences in the reported activities. With this in mind, we carried out computational modeling studies of the congeneric family of 67 naphthalene-derived compounds reported by Ratia et al. (2008); Ghosh et al. (2010), Báez-Santos et al. (2014a), Ghosh et al. (2009), providing relevant information about their binding modes and the causes of their differential activities. We assumed this information could be helpful for designing new potential PLpro inhibitors.

## 2 Materials and methods

### 2.1 Preparation of naphthalene-derived compounds

The 67 structures of naphthalene-derived compounds and their IC_50_ values were collected from references of Ratia et al. (2008), Ghosh et al. (2010), Báez-Santos et al. (2014a), Ghosh et al. (2009). The chemical structures for each compound are in Table 1. Each compound has a name formed by the letters A, B, C, and D to differentiate the article of origin, followed by the compound identification in the article (compounds from references (Ratia et al., 2008; Ghosh et al., 2010; Báez-Santos et al., 2014a), and (Ghosh et al., 2009) are named A_x, B_x, C_x, and D_x, respectively). Table 1 represents a set of 24 inhibitors (compounds A_x and D_x) that contain a benzamide and a set of 43 compounds (compounds B_x and C_x) that contain a piperidine ring.

**TABLE 1:** Structures and activities of naphthalene-derived compounds as SARS-CoV-1 PLpro inhibitors.

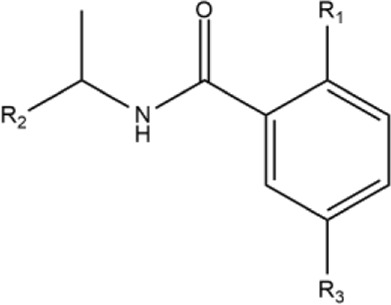						

Compound	R_1_	R_2_	R_3_	R_4_	R_5_	IC_50_ (µM)
7724772 (S)	Me	2-naphthyl	H	____	____	>200
7724772 (R)	Me	2-naphthyl	H	____	____	8.7 ± 0.7
A_3 (R)	Me	2-naphthyl	H	____	____	14.5 ± 0.9
A_4 (R)	Me	2-naphthyl	H	____	____	>200
A_5 (R)	Me	1-naphthyl	H	____	____	2.3 ± 0.1
A_6 (R)	Me	1-naphthyl	NHAc	____	____	2.6 ± 0.1
A_7 (R)	Me	1-naphthyl	NO_2_	____	____	7.3 ± 0.9
GRL0617	Me	1-naphthyl	NH_2_	____	____	0.6 ± 0.1
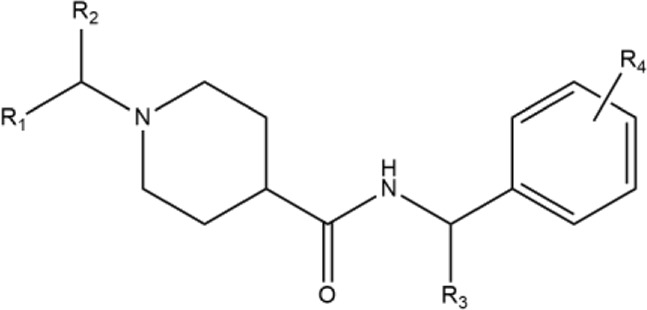						
65778771	1-naphthyl	H	H	2-OMe	____	59.2 ± 7.8
B_7a	1-naphthyl	H	H	4-OMe	____	116 ± 30
B_7b	1-naphthyl	H	H	3-OMe	____	30 ± 3
B_15a	1-naphthyl	Me	H	4-OMe	____	1.21 ± 0.04
B_15b	1-naphthyl	Me	H	3-OMe	____	0.34 ± 0.01
B_15c	1-naphthyl	Me	H	2-OMe	____	0.34 ± 0.01
B_15d	2-naphthyl	Me	H	3-OMe	____	13.2 ± 0.6
B_15e	2-naphthyl	Me	H	4-OMe	____	34.8 ± 4.0
B_15f	2-naphthyl	Me	H	3-OMe	____	5.8 ± 0.1
B_15g (R)	1-naphthyl	Me	H	3,4-O-CH_2_-O	____	0.32 ± 0.01
B_15h (S)	1-naphthyl	Me	H	3,4-O-CH_2_-O	____	0.56 ± 0.03
B_15i	1-naphthyl	H	H	3,4-O-CH_2_-O	____	∼45
B_15j	2-naphthyl	H	H	3,4-O-CH_2_-O	____	∼100
B_15k	1-naphthyl	Gem-dimethyl	H	3,4-O-CH_2_-O	____	>200
C_1a (R,S)	1-naphthyl	CH_2_Me	H	3,4-O-CH_2_-O	____	17.2 ± 0.03
C_1b (R,S)	1-naphthyl	CH_2_OH	H	3,4-O-CH_2_-O	____	32.0 ± 4.5
C_1c (R,S)	1-naphthyl	CH_2_OMe	H	3,4-O-CH_2_-O	____	>100
C_1d (R,S)	1-naphthyl	CH_2_Ph	H	3,4-O-CH_2_-O	____	>100
C_2a (R)	1-naphthyl	Me	H	H	____	2.2 ± 0.1
C_2b (R)	1-naphthyl	Me	(R)-Me	H	____	13.5 ± 1.2
C_2c (R)	1-naphthyl	Me	(S)-Me	H	____	12.7 ± 0.3
C_2d (R)	1-naphthyl	Me	(R)-CH_2_OMe	H	____	18.0 ± 1.9
C_2e (R)	1-naphthyl	Me	(S)-CH_2_OMe	H	____	1.9 ± 0.1
C_3a (R)	1-naphthyl	Me	H	4-Et	____	0.47 ± 0.01
C_3b (R)	1-naphthyl	Me	H	4-CO-NH-Me	____	0.60 ± 0.02
C_3c (R)	1-naphthyl	Me	H	3-CO-NH-Me	____	0.63 ± 0.01
C_3d (R)	1-naphthyl	Me	H	4-NH-CO-Me	____	5.7 ± 0.5
C_3e (R)	1-naphthyl	Me	H	3-NH-CO-Me	____	0.39 ± 0.01
C_3f (R)	1-naphthyl	Me	H	3-CH_2_-NH-CO-Me	____	20.4 ± 1.2
C_3g (R)	1-naphthyl	Me	H	3-Cl	____	27.2 ± 4.1
C_3h (R)	1-naphthyl	Me	H	4-Cl	____	0.58 ± 0.02
C_3i (R)	1-naphthyl	Me	H	3,4-diF	____	29.2 ± 2.1
C_3j (R)	1-naphthyl	Me	H	4-F	____	0.49 ± 0.01
C_3k (R)	1-naphthyl	Me	H	3-F	____	0.15 ± 0.01
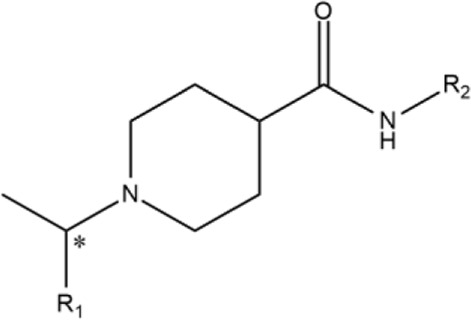						
C_4a (R,S)	8-quinolinyl	3-F-Ph-CH_2_	____	____	____	7.0 ± 0.7
C_4b (R,S)	5-quinolinyl	3-F-Ph-CH_2_	____	____	____	4.5 ± 0.2
C_4c (R,S)	5-isoquinolinyl	3-F-Ph-CH2	____	____	____	6.8 ± 0.3
C_4d (R,S)	1-isoquinolinyl	3-F-Ph-CH_2_	____	____	____	30.8 ± 2.6
C_5a (R)	1-naphthyl	3-pyridinyl-CH_2_	____	____	____	26.3 ± 2.3
C_5b (R)	1-naphthyl	4-pyridinyl-CH_2_	____	____	____	18.3 ± 0.9
C_5c (R)	1-naphthyl	2-methoxy-4-pyridinyl-CH_2_	____	____	____	0.35 ± 0.02
C_6a (R)	1-naphthyl	4-Cl-Ph-CH_2_CH_2_	____	____	____	1.6 ± 0.3
C_6b (R)	1-naphthyl	3-F-Ph-CH_2_CH_2_	____	____	____	1.9 ± 0.1
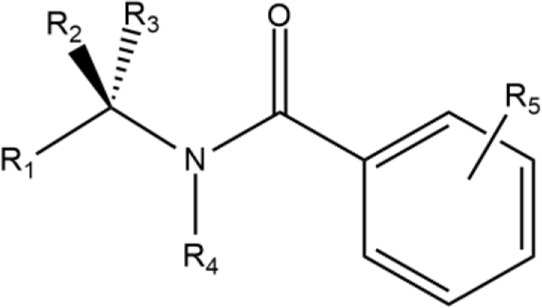						
D_2	1-naphthyl	Me	H	H	2-Me and 5-CH_2_NH_2_	0.46 ± 0.03
D_5a	2-naphthyl	Me	H	H	3-Me	14.8 ± 5.0
D_5b	2-naphthyl	Me	H	H	4-Me	29.1 ± 3.8
D_5c	2-naphthyl	Me	H	H	2-OMe	90 ± 26
D_5d	2-naphthyl	Me	H	H	3-OMe	13.5 ± 6.8
D_5e	2-naphthyl	Me	H	H	4-OMe	149 ± 43
D_5f	2-naphthyl	Me	H	H	2,6-diMe	12.1 ± 0.7
D_9	2-naphthyl	Me	H	H	4-NH_2_	46.1 ± 13.0
D_21	1-naphthyl	Me	H	Me	2-Me	22.6 ± 6.9
D_23	1-naphthyl	Me	H	H	4-NH_2_	24.8 ± 1.0
D_29	1-naphthyl	Me	Me	H	2-Me and 5-NH_2_	11.1 ± 1.3
D_33	1-naphthyl	Me	H	H	2-Me and 5-CN	5.2 ± 0.5
D_40	1-naphthyl	Me	H	H	2-CH_2_OMe and 5-NH_2_	2.7 ± 0.1
D_32	1-naphthyl	Me	H	H	2-Me and 5-I	1.4 ± 0.3
D_47	1-naphthyl	Me	H	H	2-Me and 5-CH_2_NHBoc	4.8 ± 0.4
D_49	1-naphthyl	Me	H	H	2-Me and 5-CH_2_NHMe	1.3 ± 0.1

The structures were drawn in Maestro Molecular Editor (Maestro 12.8.117, Schrödinger LLC, New York, NY, USA, 2021) and processed using the Maestro’s module LigPrep. The protonation states were estimated using Epik (Shelley et al., 2007) under a physiological pH value of 7. In the case of compounds containing two possible enantiomers or presented in racemic form, both were chosen for molecular docking experiments to explore interactions at the PLpro binding site.

### 2.2 Preparation of SARS-CoV-1 PLpro structures

The three-dimensional (3D) crystallographic structures of the SARS-CoV-1 PLpro were obtained from the Protein Data Bank (PDB). We selected those structures co-crystallized with non-covalent inhibitors derived from naphthalene in the S_3_ and S_4_ sub-sites of the protease. Four PLpro-ligand structures were selected with the PDB IDs 3E9S (with GRL0617, resolution 2.50 Å) (Ratia et al., 2008), 3MJ5 (with B_15g, resolution 2.63 Å) (Ghosh et al., 2010), 4OVZ (with C_3j, resolution 2.50 Å), and 4OW0 (with C_3k, resolution 2.10 Å) (Báez-Santos et al., 2014a). The Protein Preparation Wizard (Schrödinger LLC, New York, NY, USA, 2021) was used to improve PDB models. Missing atoms were assigned, and hydrogen atoms were added to have all the atoms represented and positioned explicitly. Crystallographic water molecules were removed, and native zinc ions were retained. Hydrogen bonding networks were optimized by reorienting hydroxyl groups, thiol groups, asparagine and glutamine amide groups, and histidine imidazole rings. Predictions of the protonation states of the ionizable groups were performed. Finally, the structures were minimized using the OPLS force field (Harder et al., 2016).

Given the conformational diversity of the binding site of the naphthalene derivatives, we performed a previous analysis of the structures to know in more detail about the flexibility of the binding site of these compounds. For that, we aligned the structures coded 3MJ5, 4OVZ, and 4OW0 with the 3E9S structure and compared the orientation of the residues distributed at 5 Å from the ligand with root-mean-square deviation (RMSD) calculations (details in the Supplementary Table S1). This information was used as material for the development of subsequent analyses. In the comparison between structures, very little variation was observed in the conformations of the active site residues. Two orientations for Gln270 were observed: the first orientation at 3E9S and the second orientation at 3MJ5, 4OVZ, and 4OW0. The remaining residues did not show significant differences among them. Therefore, we selected 3E9S and 4OW0 (the latter having a better resolution than its analogues) to perform the docking calculations.

### 2.3 Docking calculations

Ligand-receptor docking calculations were performed using Glide from the Schrödinger suite to obtain binding modes (Friesner et al., 2004). The ligand array was docked inside the protein binding site using a 20 Å × 20 Å x 20 Å grid centered on residues corresponding to PLpro subsites S_3_ and S_4_. Glide standard (SP) and extra precision (XP) modules were used. Glide SP is a more indulgent function and allows the identification of ligands with a reasonable tendency to bind. On the other hand, the extra precision module (XP) is a more strict function, which penalizes poses that violate physical-chemistry principles (Friesner et al., 2006). Using these modules together allowed access to good quality solutions. Glide SP was used to evaluate the ability of the protocol to find poses with similar interactions to those present in the crystallographic structures; meanwhile, the less indulgent XP function was used to obtain the final docking poses, which were used to start the analysis.

Default settings were used, where a flexible ligand was sampled in a rigid protein. Firstly, conformers were generated for each ligand. During this process, ring conformations were discarded if their energies were higher than that of the lowest conformation by more than 2.5 kcal/mol. No more than 5000 poses per ligand were selected to pass to the grid refinement calculation. The rough-score cutoff (relative to the best rough score accumulated so far) for keeping poses for refinement was 100. Then, at most 400 poses (in SP) or 800 poses (in XP) per ligand were kept for energy minimization. During minimization, the distance-dependent dielectric constant setting was 2.0, and the maximum number of minimization steps (conjugate gradient minimization algorithm) was 100. The best five poses were considered for selecting the best pose.

The best pose for each ligand was chosen by employing two criteria. The first one corresponds to a score-based criterion, where the Emodel score was considered to find the best pose for a given ligand and the GlideScore to rank compounds based on their binding to the receptor. After this, an interaction-based criterion was considered, i.e., we selected poses that present interactions similar to that of the co-crystallized naphthalene-derived compounds.

### 2.4 LigRMSD

When docking congeneric compounds, we expect the binding mode to be conserved with respect to those of co-crystallized compounds in the PLpro structures selected for this study. Therefore, we compared the binding poses obtained by molecular docking calculations using the LigRMSD web server (Velázquez-Libera et al., 2020). LigRMSD allows selecting the maximum common substructure between the molecules being compared, establishing matching graphs between them, and calculating the RMSD between the equivalent atoms with respect to the reference. The match is defined using the values “%Ref” and “%Mol”. “%Ref” indicates the percentage of common graphs between a docked compound and a selected reference, related to the total number of atoms of the selected reference. On the other hand, “%Mol” is the percentage of common graphs between the docked compound and the selected reference, with respect to the total number of atoms of the docked compound. These values obtained from the LigRMSD server represent the maximum similarity between the compounds being compared, so high values of “%Ref” and “%Mol” are associated with high similarity between the compared compounds.

Based on this, we compared the poses obtained using multiple references. The poses of the co-crystallized ligand GRL0617 and 6577871 were used as references for compounds docked inside the PDB with code 3E9S. In addition, the poses of the co-crystallized compound C_3k and 7724772 were used as references for compounds docked inside the PDB with code 4OW0.

### 2.5 Interaction fingerprint (IFP)

Recurrent chemical interactions between the docked poses of ligands and residues in the SARS-CoV-1 PLpro binding site were captured by Interaction fingerprints (IFPs) (Deng et al., 2004). Maestro’s Interaction Fingerprint panel was used to build them. This method describes the presence or absence of chemical interactions between ligands and binding residues using bits for the subsequent construction of an interaction matrix. Each bit describes if a specific type of interaction takes place between the ligand and a protein residue, considering hydrophobic (H), polar (P), and aromatic (Ar) interactions. It is also possible to detect whether a residue is acting as a hydrogen bond (HB) acceptor (A) or donor (D) and electrostatic interactions with charged groups (Ch). For this study, it was counted as an interaction when a PLpro residue is within a maximum cutoff distance of 4.0 Å between the heavy atoms with respect to the ligand atoms.

### 2.6 Gaussian accelerated molecular dynamics (GaMD) and correlation analysis

Molecular dynamics (MD) simulations were performed to obtain a diverse sampling of the SARS-CoV-1 PLpro binding site. They had to be carried out with ligands at the binding site to ensure that the site remained open, allowing for the inclusion of other ligands in the subsequent cross-docking calculations. When placing a ligand, induce-fit effects may occur due to a specific ligand. To mitigate the induced effects resulting from a single ligand, the PDB protein structures with codes 3E9S and 4OW0, complexed with the ligands GRL0617 and C_3k, were used to generate four PLpro-ligand models (in the case of the structure with code 4OW0, only the first chain was used). Two of these models were the original structures 3E9S and 4OW0, containing the ligands GRL0617 and C_3k, respectively. The other two models were the structures previously obtained by docking 3E9S with C_3k and 4OW0 with GRL0617. This approach aimed to introduce greater variation in the starting structures.

Protein structures were prepared using the Protein Preparation Wizard (Schrödinger LLC, New York, NY, USA, 2021). From this, force field parameters and coordinate files were constructed using LEAP from Amber (Case et al., 2005). A regular truncated octahedral TIP3P water box with 12 Å between the solute and the edges of the box was used for the simulations. The system minimization was carried out for 10,000 steps. Two rounds of equilibration were then performed. The system was heated to 310K for 1 ns using an isothermal-isovolumetric (NVT) assembly, followed by an isothermal-isobaric (NPT) equilibration for 80 ns.

To perform Gaussian accelerated molecular dynamics (GaMD) (Miao and McCammon, 2017), the pmemd.cuda implementation of Amber20 was used to generate four trajectories. We used the LiGaMD method (Miao et al., 2020), based on GaMD, which was necessary for more efficient sampling simulations of protein-ligand complexes’ binding and unbinding process. First, a 60-ns MD simulation was performed. The first 10 ns correspond to a conventional preparatory MD, without statistical collection, followed by 50 ns of LiGaMD. Next, a production simulation was performed, which starts at 50 ns and extends up to 150 ns. The VMD (Humphrey et al., 1996) and CPPTRAJ (Roe and Cheatham, 2013) tools were used to analyze the trajectories.

The trajectories generated for all systems were grouped using the K-means participle algorithm to obtain greater conformational diversity. An internal script using the scikit-learn library (Varoquaux et al., 2015) was used to perform the protocol. The different clusters were obtained considering six distance descriptors; (a) RMSD value of the Q270 residue; (b) distance between the more proximal carboxylate oxygen of the side chain of D165 and the nitrogen at the side chain of Q270; (c) distance between the hydroxyl group of Y269 and the nitrogen of the side chain of Q270; (d) distance between the nitrogen in the side chain of K158 and the oxygen at the side chain of Q270; (e) distance between the backbone oxygen of residue N268 and the nitrogen of C271, and (f) distance between the hydroxyl group of Y265 and the oxygen backbone of N268. Based on this, the possible clusters were represented by a dendogram or “cluster tree,” where the root corresponds to the largest cluster containing all the sampled states, and each leaf refers to a single cluster.

The clustering process allowed us to find representative protein structures from the trajectories. The obtained protein structures were used as receptors of molecular cross-docking with each of the compounds under study, resulting in different poses for each ligand. The same docking settings described in Section 2.3 were employed for cross-docking. An *in-house* Python script (Muñoz-Gutierrez et al., 2016) was used to select a representative complex for each ligand to best fit the correlations between the energy values calculated from the docking process and the logarithmic activities of the series of naphthalene-derived compounds. The result of the protocol corresponds to protein-ligand complexes showing the highest correlations.

## 3 Results and discussion

### 3.1 Docking predictions

**FIGURE 1:**
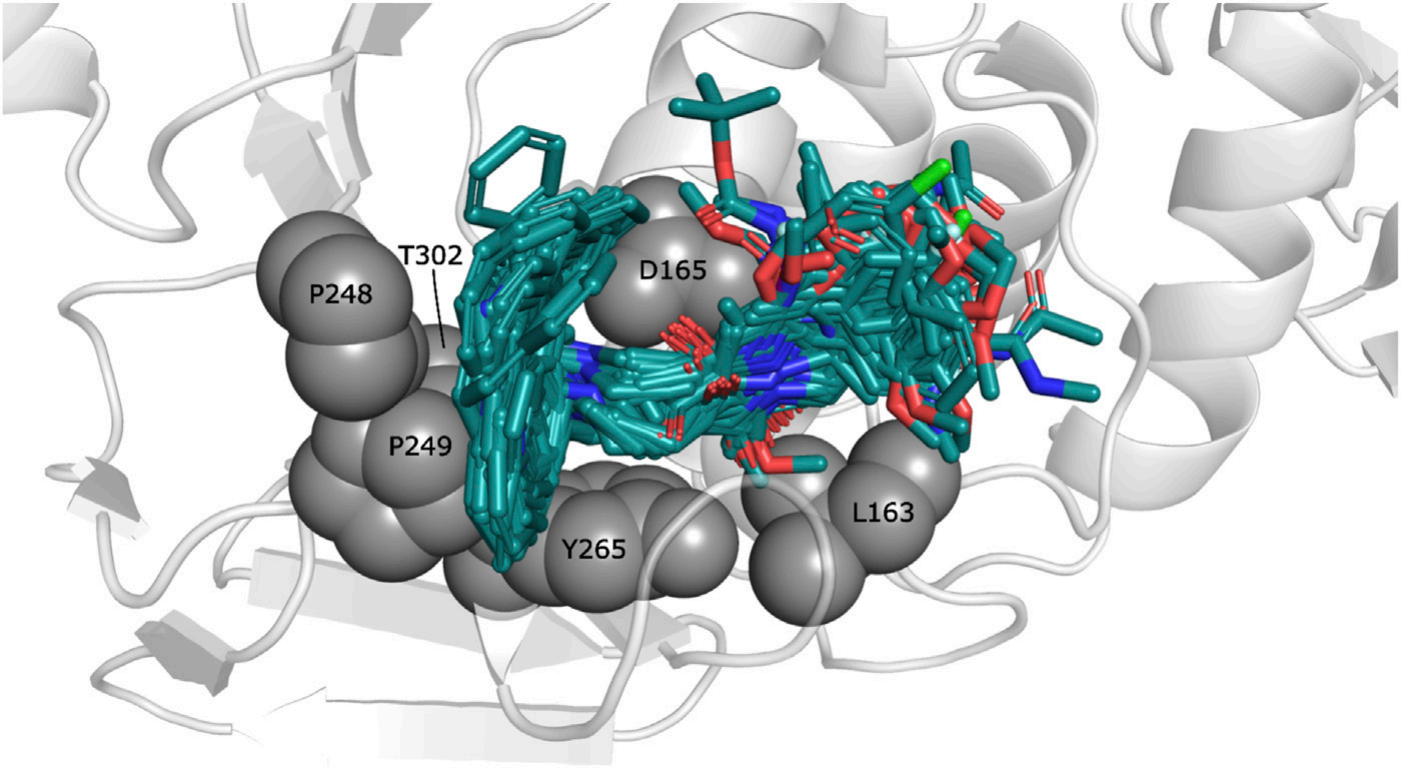
Docked structures within the SARS-CoV-1 PLpro binding site. Docked ligands are represented by sticks. Relevant residues at S_3_ and S_4_ subsites are represented by spheres.

The ligands were docked to study the molecular basis of the interactions between the naphthalene-derived compounds and the SARS-CoV-1 PLpro (docking scoring energies are reported in the Supplementary Table S2). It can be seen that all ligands in the series adopt the same binding mode, placing the naphthylmethylamine group at the S_4_ subsite of the enzyme (Figure 1). It has been previously verified that this subsite is specific for leucine and can accommodate large hydrophobic groups (Rut et al., 2020). Olsen et al. observed that the 2-benzothiazolyl and (4-hydroxyphenyl)ethyl groups of the covalent inhibitors VIR250 and VIR251 occupy opposite sides of the broad S_4_ pocket of SARS-CoV-1 and SARS-CoV-2 PLpro (Rut et al., 2020; Patchett et al., 2021); chemical groups at S_4_ can be oriented closer to the Pro249 or closer to the Pro248. Our docking results show that the naphthylmethylamine group can occupy both sides of the S_4_ subsite.

**FIGURE 2:**
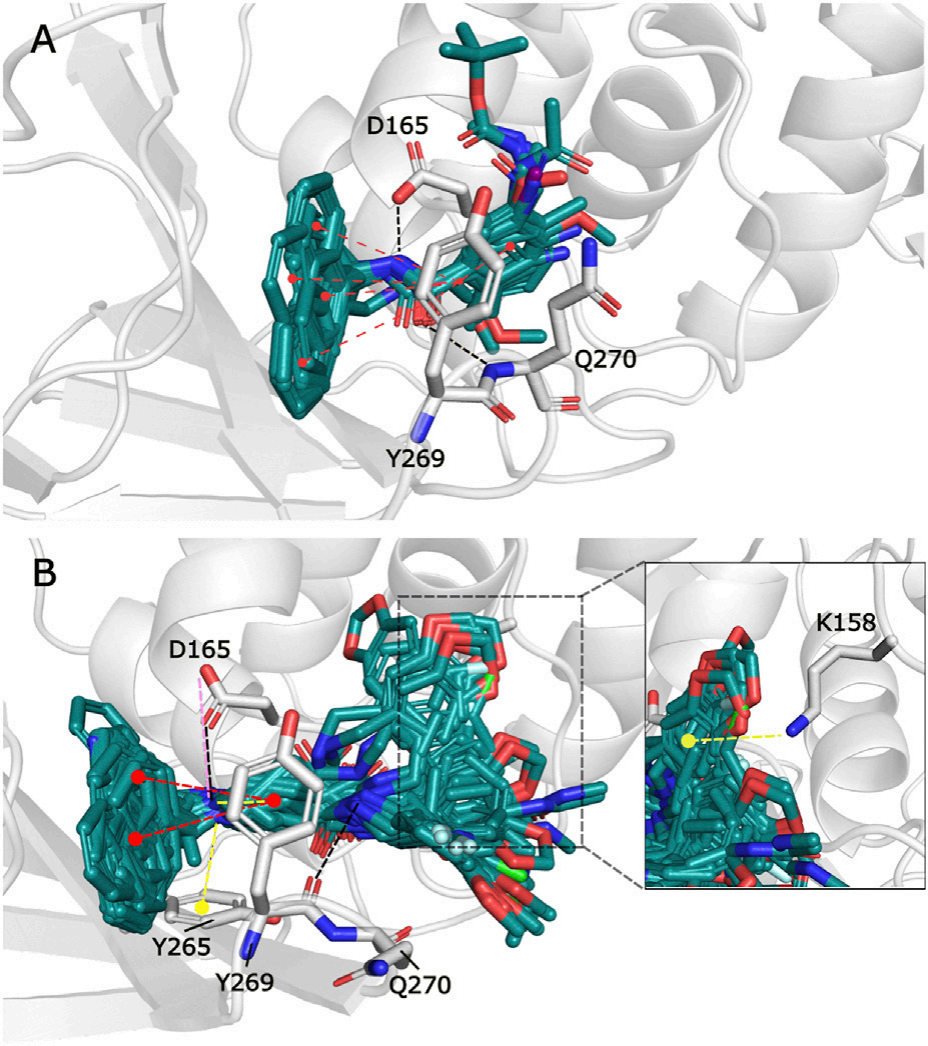
Docking poses for congeneric series of naphthalene-derived compounds at the SARS-CoV-1 PLpro binding site. **(A)** Compounds from series A, D, 7724772, and GRL0617 docked inside the structure with PDB code 3E9S. **(B)** Compounds from series B, C, and 6577871 docked inside the structure with PDB code 4OW0 (a rotation of a selection is at the right to observe interactions with Lys158). Ligands are represented by cyan sticks, while protein residues involved in interactions are represented with white sticks. Interactions are represented by dashed lines with the following coloring scheme: red color lines correspond to pi-pi stacking interactions, yellow color lines to pi-cation interactions, black color lines to HBs, and magenta color lines to salt bridges.

The observed interactions are consistent with those reported for crystals having co-crystallized naphthalene-derived compounds (Ratia et al., 2008; Báez-Santos et al., 2014a). Compounds from series A, D, 7724772, and GRL0617 have HB interactions between the benzamide carbonyl of the inhibitors and the backbone NH of Gln270 (Figure 2A); the same interaction is absent in the poses obtained for compounds from series B, C, and 6577871 (Figure 2B). This occurs since the BL2 hinged loop exists in different conformations for each of the studied protein states. For the structure with PDB code 4OW0, the side chain of the Gln270 residue is moved away from the inhibitors, preventing HB formation between its backbone and compounds from series B, C, and 6577871 (Báez-Santos et al., 2014b). On the hand, the residue Tyr269 (also at the BL2 loop) does not have a considerable displacement between the structures with codes 3E9S and 4OW0 and is involved in pi-pi stacking interactions. This residue, and the residue Asp165 (forming HBs with donors of the ligands), are of great importance for stabilizing the naphthalene-derived compounds (Figure 2). Asp165 also forms a salt bridge with the protonated piperidine of compounds from series B, C, and 6577871. The aromatic group of the residue Tyr265 forms a pi-cation interaction with the same protonated piperidine groups (Figure 2B). It is also pertinent to point out that the residue Lys158 establishes pi-cation interactions with several aromatic substituents placed in its vicinity (Figure 2B to the right).

**FIGURE 3:**
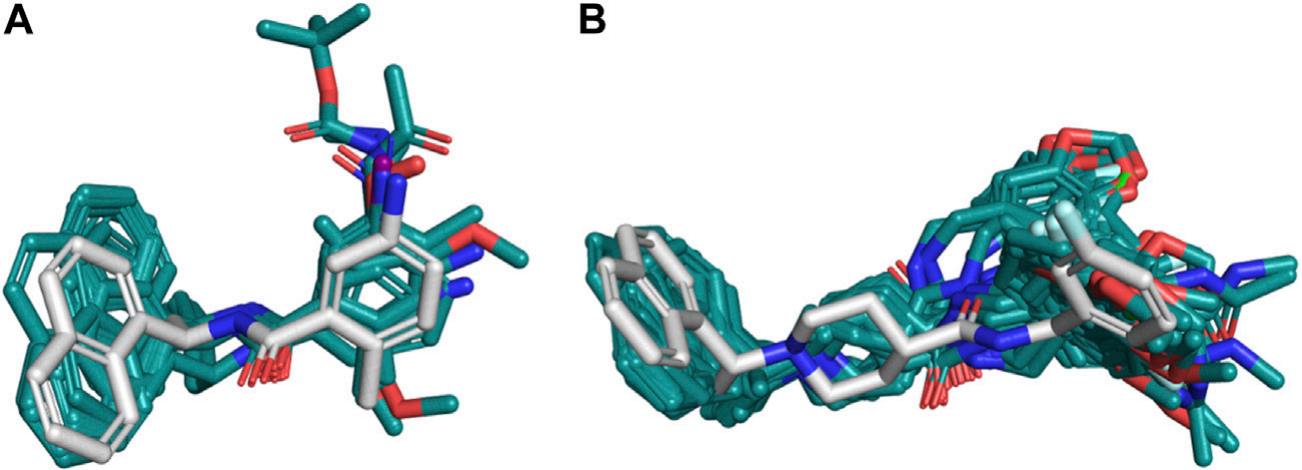
Structural similarity of the docking poses with respect to references 3E9S and 4OW0. **(A)** Compounds from series A and D compared to compound GRL0617 co-crystallized on 3E9S as reference. **(B)** Compounds from series B and C compared to compound C_3k co-crystallized on 4OW0 as reference. For each of the cases, the reference is represented as white sticks, while the poses obtained by docking are shown in cyan.

The poses obtained from the molecular docking of the 67 naphthalene-derived inhibitors were compared with their similar inhibitors GRL0617 and C_3k co-crystallized on the structures with codes 3E9S and 4OW0, respectively. This comparison was carried out using the LigRMSD server, which identifies common graphs between molecules and calculates the RMSD between the equivalent atoms in each graph (Velázquez-Libera et al., 2020). It is accepted in the literature that RMSD values less than 2 Å reflect a meaningful spatial relationship between the compared structures (Warren et al., 2006; Plewczynski et al., 2011; Sasmal et al., 2020). The results of this analysis are detailed in the Supplementary Table S3. The comparisons where the co-crystallized inhibitor GRL0617 in 3E9S was used as reference helped to characterize the orientations of the ligands from series A and D. When GRL0617 is used as reference, these compounds exhibited %Ref values higher than 85%, likewise the %Mol values in most of the cases (except for compound D_47 with %Mol = 70.97). Most RMSD values in the range of 0.25 Å to 1.5 Å were obtained, with only five compounds (A_6, D_21, D_33, D_40, and the redocked conformation of GRL0617) showing RMSD values between 2.30 and 2.51 Å. The naphthalene groups in these five compounds were positioned opposite to the same group in the reference, but their main scaffolds were oriented correctly. Therefore, docking poses of the complete set of ligands from series A and D were oriented similarly to the co-crystallized compound GRL0617. On the other hand, compound C_3k, co-crystallized in 4OW0, was used as a reference to characterize the orientations of the ligands from series B and C. When C_3k is used as reference, these compounds exhibited %Ref values higher than 84%, likewise the %Mol values in most of the cases (except for compound C_1d with %Mol = 75.68). As an exception, compounds C_6a and C_6b have %Ref and %Mol values of 75.86 and 73.33 respectively; these values also indicate that there is similarity with C_3k. Compounds from series B had RMSD values < 2 Å with only the compound B_15j showing RMSD = 4.04 Å. The majority of compounds from series C had RMSD values < 2 Å; however, six of the non-optically active compounds (C_2d, C_3i, C_5b, C_5c, C_6a, and C_6b) had higher values, and the optically active compounds C_1a (S), C_1b (R), C_1c (R), C_1d (R and S), C_4a (R and S), C_4c (R), and C_4d (R) have also RMSD values > 2 Å. In some cases, the aromatic groups from the benzylamine (or similar substituents in C_5a-c and C_6a-b) of the studied compounds from series B and C, rotated in the opposite direction with respect to the reference, inducing pi-cation interactions with Lys158 (Figure 2B to the right). This greater flexibility led to higher RMSD values; however, a visual analysis shows that a similar orientations with respect to the reference compound were obtained, which was reflected in the coincidence between the scaffolds of the compared compounds (Figure 3).

**FIGURE 4:**
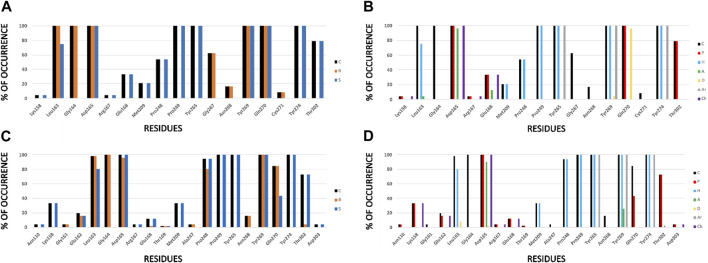
IFPs that describe interactions between docked compounds and SARS-CoV-1 PLpro crystals. **(A, B)** Interactions of compounds from series A, D, 7724772, and GRL0617 with residues at the PLpro crystal with code 3E9S. **(C, D)** Interactions of compounds from series B, C, and 6577871 with residues at the PLpro crystal with code 4OW0. Interactions in the graphs at the left **(A, C)** are presented as percentage of occurrence of contacts [C], interactions with the backbone of the residue [B], and interactions with the side chain of the residue [S]. Interactions in the graphs at the right **(B, D)** are presented as percentage of occurrence of chemical interactions: contacts [C], polar [P], hydrophobic [H], HBs where the residue is an acceptor [A], HBs where the residue is a donor [D], aromatic [Ar], and electrostatic with charged groups [Ch].

For a better understanding of the interactions between the docked ligands and PLpro, an IFP was performed. This analysis allows annotating the recurrent chemical interactions observed between the compounds of the congenic series and the protease binding site. The graphs of the types of chemical interactions occurring per residue are reported. The IFPs for the 24 compounds from series A, D, 7724772, and GRL0617 docked in the PLpro crystal with code 3E9S are in Figures 4A, B, and the IFPs for the 43 compounds from series B, C, and 6577871 docked in the PLpro crystal with code 4OW0 are in Figures 4C, D.

For both protein crystal structures, the residues implicated in the formation of interactions at the protein-ligand interface are similar (Figure 4). Hydrophobic contributions and aromatic contacts with residues Tyr265, Tyr269, and Tyr274 occur in 100% of the docked structures. These residues form an aromatic box that contribute to attraction and stabilization of the naphthalene-derived inhibitors; specifically, Tyr269 is essential for closing the BL2 loop to adopt the closed conformation of the binding site (Báez-Santos et al., 2014b). IFPs show that Tyr269 was also identified as an HB donor with ∼5% of compounds from series A and D, and as an HB acceptor with ∼25% of compounds from series B and C. These roles can be present when including substituents with specific polar groups (Figure 2).

The residues Pro248 and Pro249 favored the occurrence of hydrophobic contacts at the protein-ligand interface. Hydrophobic contacts of Pro249 had 100% of occurrence, while Pro248 also had high hydrophobic contributions, with ∼55% and ∼90% of occurrence in the structures 3E9S and 4OW0, respectively. Several residues were also identified that contributed to form electrostatic interactions at the SARS-CoV-1 PLpro binding site. Asp165 has polar interactions with the docked poses with 100% of occurrence. This residue acts as HB acceptor with more than 90% of occurrence in 3E9S and 4OW0, respectively. It reflects that this residue forms HBs with benzamide NH group of compounds from series A and D, and also forms HBs (and salt bridges) with the protonated piperidine of compounds from series B and C (Figure 2). The residue Gln270 from the BL2 loop had 100% of occurrence of polar contacts and is an HB donor in ∼95% of the docked compounds in 3E9S. It had ∼40% of occurrence of polar contacts when forming complexes between ligands and the structure with code 4OW0.

Other noteworthy IFPs are detailed as followed. Gly164 had contacts with 100% of occurrence in 3E9S and 4OW0. Leu163, its backbone, had contacts with all the ligands, and its side chain had hydrophobic interactions with ∼75% and ∼80% of occurrence in 3E9S and 4OW0, respectively. Lys158 had polar and charged contributions in ∼5% of the structures docked in 3E9S, and the same contributions in ∼30% of the structures docked in 4OW0. Glu168 had polar and charged contacts with ∼30% of occurrence and acted as HB acceptor with ∼10% of occurrence in 3E9S; in contrast, it had polar and charged contacts with ∼10% of occurrence in 4OW0. Finally, Thr302 had polar contributions in ∼80% of the structures docked in 3E9S, and the same contributions in ∼70% of the structures docked in 4OW0.

The analysis presented with the IFPs shows two variants of how two sets of non-covalent inhibitors bind to the S_3_-S_5_ subsites. It is possible to observe some interactions that seem essential and others that appear occasionally. The IFPs show how substituents of the studied sets are distributed at S_4_. The naphthalene group can be oriented closer to Pro249 or in the opposite direction, closer to Pro248 (similar to the structures of complexes between PLpro with the covalent inhibitors VIR250 and VIR251) (Rut et al., 2020; Patchett et al., 2021).

### 3.2 Binding site flexibility and correlation results

In order to increase the conformational sampling of the SARS-CoV-1 PLpro binding site in the presence of naphthalene-derived inhibitors, four GaMD simulations were performed following the protocol described in the Materials and Methods section. Two of them were carried out on the solvated PDB structure with code 3E9S in complex with GRL0617 and C_3K, while the others two simulations were developed on the solvated structure with code 4OW0 coupled to the same ligands. Stability of the GaMD trajectories using the RMSD of the positions for the backbone PLpro atoms as a function of simulation time was evaluated; RMSD was reasonably stable during the production simulation for all the systems (Supplementary Figure S2).

From the GaMD simulations, six distance descriptors (Materials and Methods section) were considered to perform a partition clustering process by means of the K-means algorithm. This clustering algorithm assigns all MD conformations into one large grouping. The largest cluster was divided into two subclusters iteratively until each conformation forms a single cluster (Abramyan et al., 2016). The value of *k* in the algorithm was defined using the “elbow method” as well as a dendrogram or cluster tree plot, thus confirming that the data set contains five clusters (Shi et al., 2021). This process, applied to four GaMD simulations, resulted in twenty representative and structurally diverse PLpro conformations, named c0-c19 in this manuscript (c0-c9 and c10-c19 were derived from GaMD simulations of the models constructed from structures with codes 3E9S and 4OW0, respectively). These structures were used to perform the cross-docking methodology (67 compounds were docked in twenty PLpro structures with diverse conformations of the binding site). It is important to remark that significative variations were identified in the binding sites for c0-c19, mainly in the BL2 loop (Supplementary Figure S3).

The cross-docking yielded twenty different poses for each ligand. The orientations of these poses were verified with LigRMSD (Velázquez-Libera et al., 2020) to ensure the presence of reasonable solutions. Representative PLpro-inhibitor complexes for each ligand were selected after application of the *in-house* Python script (Muñoz-Gutierrez et al., 2016) that optimize correlations between the calculated and experimental activities. This script yielded the set of PLpro-inhibitor complexes that produce the best correlation between the docking scoring energies and experimental PLpro inhibitory activities (scoring energies for the representative complexes are reported in the Supplementary Table S2).

**FIGURE 5:**
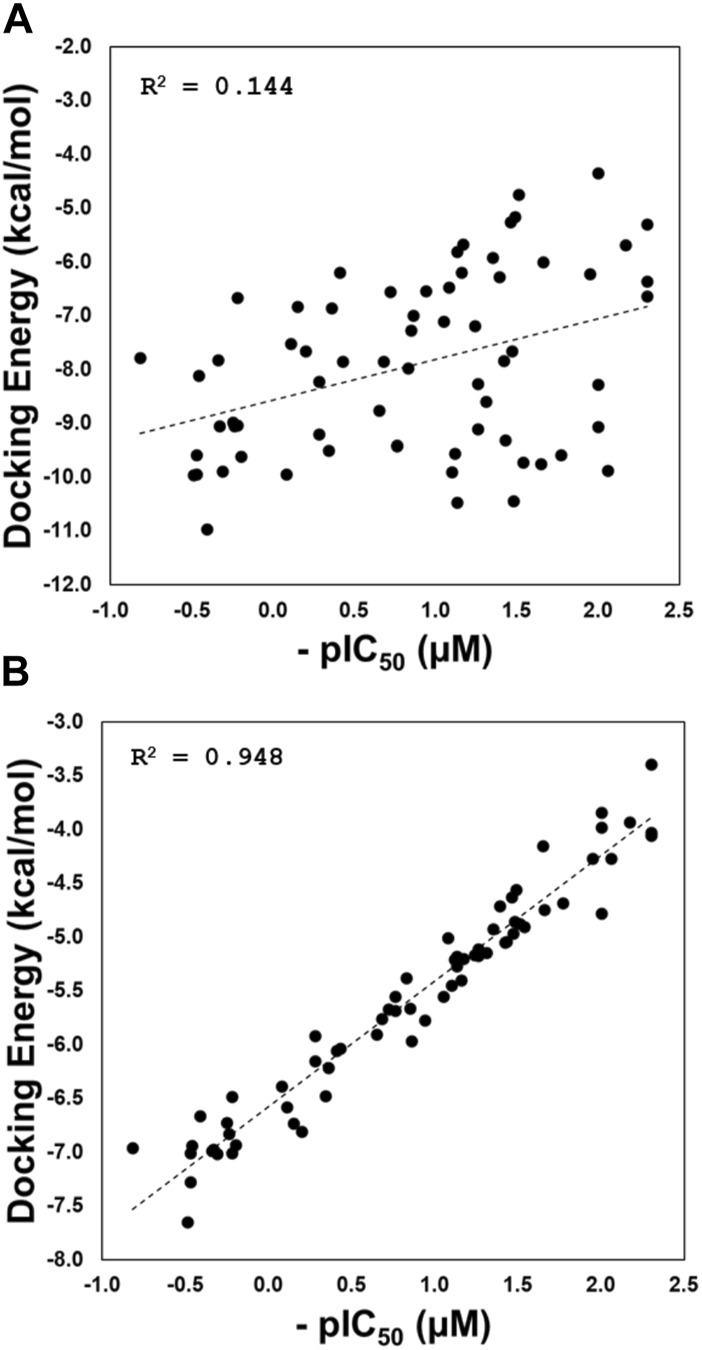
Regression plots of the docking scoring energies versus experimental activities (pIC_50_) for the docking experiments performed in structures with codes 3E9S and 4OW0 **(A)**, and for the cross-docking protocol **(B)**.

The results for correlations are depicted in Figure 5. The correlation considering the docking experiments performed in these structures with codes 3E9S and 4OW0 is poor (*R* ^2^ = 0.144; Figure 5A). This result is expected. It is well-known in literature that current docking scoring functions such as GlideScore have demonstrated satisfactory performance in docking and screening power tests; however, these functions may not be as effective when it comes to evaluating scoring power, which reflects the ability to establish a strong linear correlation between predicted and experimental binding affinities (Ferrara et al., 2004; Plewczynski et al., 2011; Su et al., 2019). To address this issue, one approach is to incorporate a flexible receptor binding site (Baumgartner and Evans, 2018). Our script employs various conformational states obtained through GaMD simulations, which allows for flexibility in the binding site. As demonstrated in Figure 5B, our method has significantly improved the correlation between predicted and experimental binding affinities, achieving an *R* ^2^ value of 0.948.

The high correlation reflects a successful explanation of the structure-activity relationship through the proposed protocol. Eleven of the twenty PLpro conformations were selected by the model, these conformations are listed in Table 2. This table also shows the list of compounds docked in each PLpro conformation to obtain the structure-activity relationship model with the highest *R* ^2^ value.

**TABLE 2:** List of structures used as receptors for cross-docking experiments and molecules involved in the structure-activity relationship model with the highest *R* ^2^.

Model	Conformation	Ligands	Model	Conformation	Ligands
3E9S	c2	B_7b, B_15j, B_15k, C_2c, C_3d, C_4d	4OW0	c13	B_7a, B_15i, C_1c, C_1d, D_21, D_23
	c3	B_15d, C_1b, D_32		c15	7724772(R), B_15b, C_3h, C_3j, C_4a, C_4b, D_5a, D_5f
	c4	C_1a, C_2a, C_3g, C_3i, C_5b, D_2		c16	A_7, B_15h, C_4c, D_5b, D_29, D_33, D_47
	c6	7724772(S), A_4, C_2b, D_5c		c17	A_6, C_3b, C_3k, C_5c, C_6a, D_40, GRL0617
	c8	6577871, A_3, B_15e, C_2d, C_3f, D_9		c19	A_5, B_15c, B_15g, C_2e, C_3a, C_3c, C_6b, D_5d, D_49
	c9	B_15a, B_15f, C_3e, C_5a, D_5e			

The GaMD and clustering process was performed to obtain different conformations of the SARS-CoV-1 PLpro binding site, and this was achieved mainly due to large changes in the BL2 loop (Supplementary Figure S3). Different versions of the binding site were obtained, which in turn differ from the binding sites in the PDB structures coded 4OW0 and 3E9S. There are some differences in the BL2 loop when comparing the 4OW0 and 3E9S structures. The residues Tyr269 and Gln270 adopt different conformations between these structures, representing a more opened (4OW0) and more closed (3E9S) state of the BL2 loop (Supplementary Figure S4). The MD and clustering protocol produced other binding site variation options, increasing flexibility, and creating new structural conformations that were a starting point for the cross-docking calculations. The structural conformations c0-c19 have differences in the SARS-CoV-1 PLpro binding site. The analysis of the conformational variations observed for the residues that constitute this site is reported in the Supplementary Table S4. From this table, it can be seen that most of the structural units being compared have RMSD values greater than 2.0Å, reflecting displacements between the parts being compared. In some cases, these variations are related to specific fluctuations that do not reflect the fluctuations of the macromolecule or the portion being compared as a whole. Consequently, a root mean square fluctuation (RMSF) analysis was performed considering the residues that are part of the binding site (Supplementary Figure S5). RMSF shows that the BL2 loop residues Tyr269 and Gln270 are the most mobile residues within the binding site. Therefore, RMSD analyses were performed on these residues (Supplementary Table S5). It is observed that most of the structures presented RMSD values higher than 2.0Å reflecting the conformational diversity of the BL2 loop between the conformations c0-c19. The high RMSD values in this part of the binding site reflect the possibility of great flexibility that justify the use of our GaMD and clustering protocol, instead of the rigid structures coming from PDB.

**FIGURE 6:**
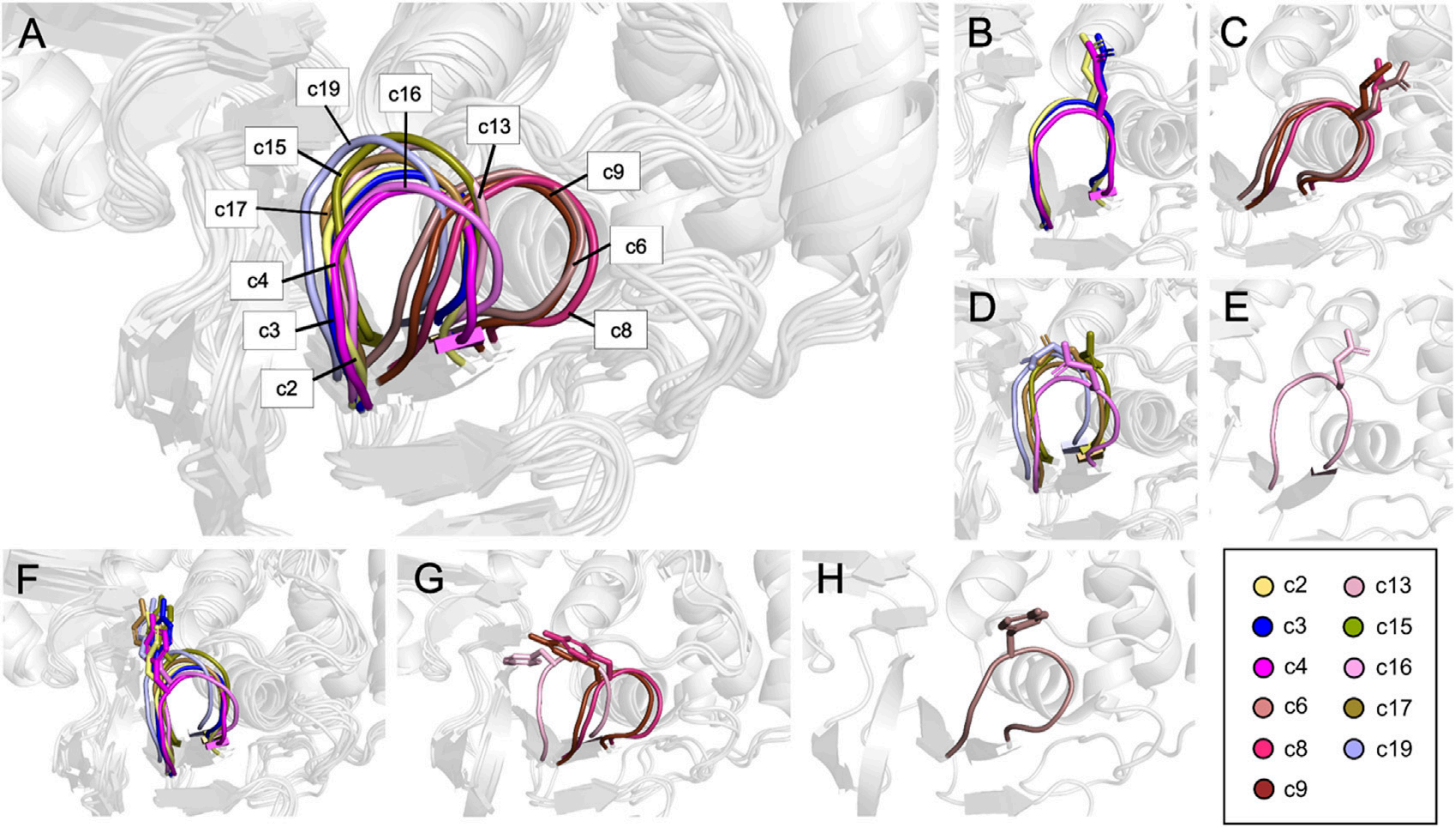
Residues conforming the BL2 loop in different clusters. **(A)** Representation of the BL2 loop for the 11 structures that maximize the structure-activity correlation. **(B)** Conformation I for Q270. **(C)** Conformation II for Q270. **(D)** Conformation III for Q270. **(E)** Conformation IV for Q270. **(F)** Conformation I for Y269. **(G)** Conformation II for Y269. **(H)** Conformation III for Y269.

From the twenty conformations c0-c19, eleven participated in the model that maximizes the structure-activity correlation, when six and five were derived from the 3E9S and 4OW0 structures, respectively. RMSD analyses for these eleven conformations were performed considering the residues with the highest fluctuations (Tyr269 and Gln270) in the binding site (using values in Supplementary Table S5), and high RMSD values for most cases were observed. The Figure 6 shows a visual inspection of the residues Tyr269 and Gln270 in the eleven conformations that are in the model that maximizes the structure-activity correlation. Gln270 presents four different orientations named I, II, III, and IV (Figures 6B–E), while Tyr269 can be grouped in three different orientations named I, II, and III (represented in the Figures 6F–H). The remaining residues at the binding site do not present considerable changes. Three structures (c2, c3, and c4) adopted conformation I for Tyr269 and I for Gln270, including 15 inhibitors. Four structures (c15, c16, c17, and c19) adopted conformation I for Tyr269 and III for Gln270, including 31 inhibitors. Two structures (c8 and c9) adopted conformation II for Tyr269 and II for Gln270, including 11 inhibitors. The combination of conformations II of Gln270 and III of Tyr269 was present in c6 that contains 4 inhibitors, and the combination of conformations IV of Gln270 and II of Tyr269 was present in c13 that contains 6 inhibitors. In all cases, the ligand poses included in the model with the highest *R* ^2^ value had the expected interactions with the residues corresponding to the BL2 loop. A visual analysis shows that the complexes in this model share interaction profiles similar to each other and concordant with the crystallographic structures. Table 2 shows that the most active compounds (c_3k, B_15g, B_15c, and B_15b) were selected in the PLpro conformations c15, c17, and c19, which adopt conformation I for Gln270 and conformation I for Tyr269, as previously mentioned (Figures 6B, F). Consequently, these receptor conformations are proposed as the most suitable for a potential exploration of new potent compounds.

Compound interactions with PLpro binding site residues for protein-ligand complexes in the highest correlation model were verified using IFPs. Previously, the most important residues were shown in an IFP analysis made on the complexes obtained by docking. It was expected that such important residues should be maintained in the complexes obtained by cross-docking. The Supplementary Figure S6 shows that the recurrent chemical interactions between the compounds and the PLpro binding site were kept in the protein-ligand complexes present in the model with the highest correlation. The most important interactions with the residues Leu163, Gly164, Asp165, Pro248, Pro249, Tyr265, Tyr269, Gln270, Tyr274, and Thr302 previously identified, were also present in the IFPs in Supplementary Figure S6. Interestingly, both series of compounds have remarkably increased polar interactions with the side chain of Arg167 (30% of occurrence). On the other hand, compounds from series B, C, and 6577871 have remarkably increased polar interactions with the side chain of the residue Glu162 (with more than 40% of occurrence).

The high conformational variation of the two residues composing the BL2 loop implies changes in the volume and shape of the binding site, which has an influence on the specific interactions of the studied compounds. The conformational diversity in the receptor binding site contributes to the ligands adopting conformations that maximized the correlation between docking scoring and pIC_50_ values.

Our results suggest that it is very relevant to consider the flexibility of the PLpro binding site for the study of its inhibitors. The flexibility of proteins poses a significant challenge when it comes to ligand docking, as the binding site can exist in various conformations (Caballero, 2021). Docking protocols in the literature widely employ combinations of docking and MD simulations (Munoz et al., 2012; Sharma et al., 2016; Śledź and Caflisch, 2018). These methods have shown that incorporating multiple protein conformations enhances the results. For instance, Strecker and Meyer conducted a recent study in which they compared docking using several crystal structures and structures obtained from MD simulations (Strecker and Meyer, 2018). They assessed the impact of structure selection and discovered that binding site shapes not observed in any crystal structure in the PDB were accessible through 500-ns MD simulations. They demonstrated that these structures significantly contributed to accurate binding pose predictions, improved ability to distinguish active compounds (screening utility), and enhanced scoring accuracy. Our results are in agreement with what was shown in this study.

Before 2019, there were few studies on SARS-CoV-1 PLpro inhibitors using computational methods; however, some recent studies have focused on the study of SARS-CoV-2 PLpro; in some of these works, the flexibility of the PLpro binding site was studied in some way (Ferreira et al., 2022; Santos et al., 2022; Singh et al., 2022). Among the recent studies, we would like to highlight the work of Garland et al. (Garland et al., 2023). The authors virtually examined the ZINC20 database (Irwin et al., 2020) using a docking method and filtering with a pharmacophore to identify possible noncovalent PLpro modulators. Using this methodology, the authors discovered the compound VPC-300195 (IC_50_ = 15 μM). The authors found a limited diversity of active compounds, which they attributed to the rigidity of the PLpro active site in crystal structures. In part, this report proposes that the inclusion of flexibility in the binding site is necessary for future designs.

## 4 Conclusion

A set of 67 naphthalene-derived compounds as noncovalent PLpro inhibitors were studied using a flexible molecular docking protocol. In summary, the following four steps were carried out: i) the structures of the protein-ligand complexes were obtained with a rigid docking, ii) multiple conformations of the PLpro binding site were obtained by using GaMD, iii) a cross-docking was performed between the 67 compounds and selected PLpro conformations, and iv) protein-ligand complexes that represent the highest correlation between docking energies and experimental activities were selected. As a result, a set of complexes was identified where the ligands interact with a flexible binding site of PLpro. The proposed methodology proved successful, and a correlation value of *R* ^2^ = 0.948 was obtained in the aforementioned last step. Considering the flexibility of the protein by using various PDB structures and the GaMD sampling of the receptor was fundamental to achieving the proposed objective. When using a rigid docking, it is ignored that ligands can be bound with significant protein conformational changes, therefore taking into account flexibility of the binding site results in a more rational approach. Overall, the strategy employed in this article serves as a good approach to studying PLpro ligands with computational tools, and the method reflects a possible conformational selection approach. Performing a detailed structural study of the inhibitory role of naphthalene derivatives acting against the SARS-CoV-1 PLpro allows us to contribute positively to the research field aimed at the design and computational evaluation of more potent candidates against this protease.

## Data Availability

The original contributions presented in the study are included in the article/Supplementary Material, further inquiries can be directed to the corresponding author.
