# Supplementary material for: Computational study of the binding orientation and affinity of noncovalent inhibitors of the papain-like protease (PLpro) from SARS-CoV-1 considering the protein flexibility by using molecular dynamics and cross-docking
Source: Front Mol Biosci. 2023 Jun 23;10:1215499. doi: 10.3389/fmolb.2023.1215499 (PMC10326900; doi:10.3389/fmolb.2023.1215499)
Supplement: Supplementary file 1 [file Table1.DOCX]

Supplementary Material

Computational study of the binding orientation and affinity of noncovalent inhibitors of the papain-like protease (PLpro) from SARS-CoV-1 considering the protein flexibility by using molecular dynamics and cross-docking

Luis Castillo-Campos^1^, José Luis Velázquez-Libera^1^, Julio Caballero^1*^

^1^ Centro de Bioinformática, Simulación y Modelado (CBSM), Facultad de Ingeniería, Universidad de Talca, 1 Poniente No. 1141, Casilla 721, Talca 3460000 Talca, Chile


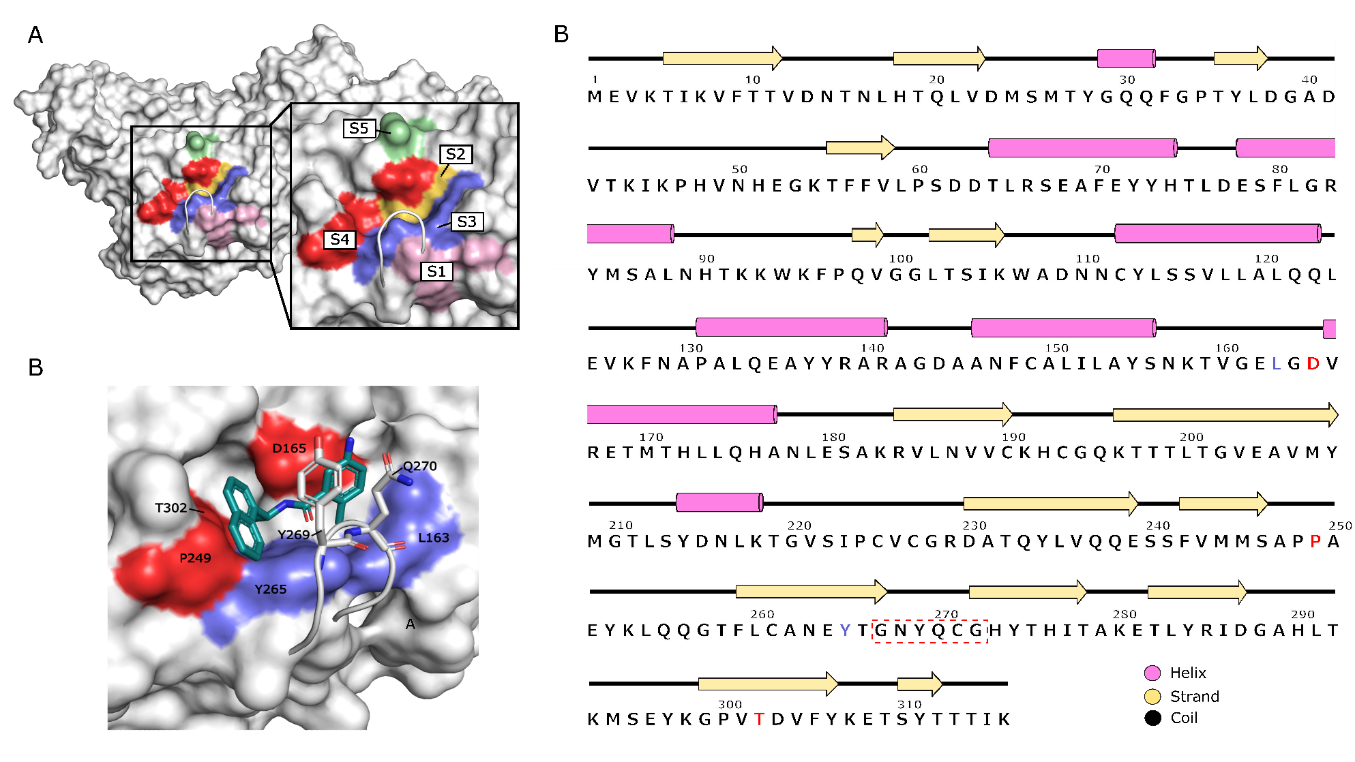


C

**Figure S1**. Subsites of SARS-CoV-1 PLpro binding site. (A) Representation as surface where the subsite S_1_ is represented in color pink, the subsite S_2_ is represented in color yellow, the subsite S_3_ is represented in color violet, the subsite S_4_ in color red, and the subsite S_5_ in green. (B) Sequence and secondary structure of SARS-CoV-1 PLpro, residues corresponding to subsite S_3_ are highlighted in violet, whereas residues of S_4_ are shown in red. The BL2 loop is in a segmented box. (C)  Binding site of naphthalene-derived compounds where the S_3_ and S_4_ sub-sites are depicted in surface; subsite S3 composed of residues Y265, L163 is colored in violet; subsite S_4_, composed of residues D165, P249 and T302 is colored in red; the BL2 loop, consisting of residues Y269 and G270, is in sticks; the ligand is presented in cyan sticks.

**Table S1**. RMSD values (Å) of SARS-CoV-1 PLpro residues within 5Å radius of the ligand atoms.^a^

| Residues | RMSD (Å) | | |
| --- | --- | --- | --- |
|  | 3MJ5 | 4OVZ | 4OW0 |
| Tyr113 | 0.22 | 0.29 | 0.34 |
| Lys158 | 1.23 | 1.27 | 1.27 |
| Leu163 | 1.41 | 1.36 | 1.44 |
| Val166 | 0.27 | 0.22 | 0.23 |
| Asp165 | **0.45** | **0.41** | **0.29** |
| Arg167 | 1.20 | 1.33 | 1.16 |
| Glu168 | 1.38 | 1.68 | 1.77 |
| Met209 | 1.52 | 1.28 | 0.86 |
| Ala247 | 0.58 | 0.81 | 0.73 |
| Pro248 | 0.99 | 1.30 | 1.27 |
| Pro249 | 1.13 | 1.12 | 1.15 |
| Tyr265 | **0.60** | **0.33** | **0.40** |
| Gly267 | 0.52 | 0.80 | 0.83 |
| Asn268 | 1.68 | 1.69 | 1.62 |
| Tyr269 | **1.04** | **1.19** | **1.04** |
| Gln270 | **5.53** | **5.06** | **5.15** |
| Cys271 | 2.41 | - | 2.48 |
| Gly272 | 0.92 | 0.82 | 0.79 |
| Tyr274 | 0.26 | 0.39 | 0.38 |
| Thr302 | 1.97 | 1.97 | 1.94 |

^a^ The PDB structure 3E9S was used as a reference. Residues causing interactions with co-crystallized ligands are highlighted in bold. Residue Cys271 corresponds to CSO271 (S-hydroxycysteine) in crystal 4OVZ, which is why the comparative value was not obtained.

**Information about Table S1**: Out of a total of 20 residues involved in the binding site of the SARS-CoV-1 PLpro protein, 90% have RMSD values below 2.0 Å. Among these residues are Asp165, Tyr265, and Tyr269, essential for forming the protein-ligand complex. However, the residue Gln270, equally important, exists in conformations other than the reference crystal 3E9S, exhibiting an RMSD > 5.0 Å. The data in the table for the 3MJ5, 4OVZ, and 4OW0 structures reveal a similarity between their binding site residues. The values for Glutamine at position 270 are 5.53 Å (3MJ5), 5.06 Å (4OVZ), and 5.15 Å (4OW0) in comparison to its position in the reference. This same residue was compared between the 3MJ5, 4OVZ, and 4OW0 structures, using the 3MJ5 crystal as a reference, resulting in RMSD values of 1.58 Å (4OVZ) and 1.53 Å (4OW0). Thus, it is possible to observe a non-significant difference in the position of Gln270. This information served as material for the subsequent analyses, which revolved around the crystallographic structures 3E9S and 4OW0, with a lower resolution (2.10 Å) compared to 3MJ5 (2.63 Å) and 4OVZ (2.50 Å).

**Table S2**. Activities of the studied compounds and their Glide scoring energies for docking performed in PDB structures with codes 3E9S or 4OW0 and representative complexes from cross-docking.^a^

| Compounds | pIC_50_ | Glide scoring energy for docking in 3E9S or 4OW0 (kcal/mol) | Glide scoring energy for representative complexes from cross-docking (kcal/mol) |
| --- | --- | --- | --- |
| **6577871** | -1.77 | -9.59 | -4.69 |
| **7724772(R)** | -0.94 | -6.55 | -5.77 |
| **7724772(S)** | -2.30 | -6.36 | -4.06 |
| **A_3** | -1.16 | -6.20 | -5.40 |
| **A_4** | -2.30 | -6.63 | -4.03 |
| **A_5** | -0.36 | -6.85 | -6.21 |
| **A_6** | -0.41 | -6.19 | -6.06 |
| **A_7** | -0.86 | -6.99 | -5.97 |
| **B_7a** | -2.06 | -9.88 | -4.27 |
| **B_7b** | -1.48 | -10.45 | -4.86 |
| **B_15a** | -0.08 | -9.95 | -6.39 |
| **B_15b** | 0.47 | -9.95 | -7.28 |
| **B_15c** | 0.47 | -9.59 | -7.01 |
| **B_15d** | -1.12 | -9.56 | -5.21 |
| **B_15e** | -1.54 | -9.73 | -4.91 |
| **B_15f** | -0.76 | -9.41 | -5.55 |
| **B_15g** | 0.49 | -9.96 | -7.65 |
| **B_15h** | 0.25 | -8.98 | -6.73 |
| **B_15i** | -1.65 | -9.75 | -4.16 |
| **B_15j** | -2.00 | -9.07 | -4.78 |
| **B_15k** | -2.30 | -5.30 | -3.40 |
| **C_1a(R)** | -1.24 | -7.19 | -5.17 |
| **C_1b(R)** | -1.51 | -4.76 | -4.88 |
| **C_1c(R)** | -2.00 | -4.35 | -3.98 |
| **C_1d(R)** | -2.00 | -8.27 | -3.85 |
| **C_2a** | -0.34 | -9.51 | -6.48 |
| **C_2b** | -1.13 | -10.47 | -5.18 |
| **C_2c** | -1.10 | -9.91 | -5.45 |
| **C_2d** | -1.26 | -9.11 | -5.18 |
| **C_2e** | -0.28 | -9.20 | -6.15 |
| **C_3a** | 0.33 | -9.05 | -6.98 |
| **C_3b** | 0.22 | -9.04 | -7.01 |
| **C_3c** | 0.20 | -9.62 | -6.93 |
| **C_3d** | -0.76 | -9.42 | -5.68 |
| **C_3e** | 0.41 | -10.96 | -6.66 |
| **C_3f** | -1.31 | -8.59 | -5.14 |
| **C_3g** | -1.43 | -9.32 | -5.04 |
| **C_3h** | 0.24 | -9.05 | -6.83 |
| **C_3i** | -1.47 | -7.65 | -4.97 |
| **C_3j** | 0.31 | -9.90 | -7.01 |
| **C_3k** | 0.82 | -7.78 | -6.96 |
| **C_4a(R)** | -0.85 | -7.27 | -5.66 |
| **C_4b(R)** | -0.65 | -8.77 | -5.91 |
| **C_4c(R)** | -0.83 | -7.97 | -5.38 |
| **C_4d(R)** | -1.49 | -5.16 | -4.56 |
| **C_5a** | -1.42 | -7.84 | -5.05 |
| **C_5b** | -1.26 | -8.27 | -5.11 |
| **C_5c** | 0.46 | -8.11 | -6.94 |
| **C_6a** | -0.20 | -7.65 | -6.81 |
| **C_6b** | -0.28 | -8.22 | -5.92 |
| **D_2** | 0.34 | -7.83 | -6.99 |
| **D_5a** | -1.17 | -5.67 | -5.20 |
| **D_5b** | -1.46 | -5.25 | -4.63 |
| **D_5c** | -1.95 | -6.22 | -4.27 |
| **D_5d** | -1.13 | -5.81 | -5.27 |
| **D_5e** | -2.17 | -5.69 | -3.93 |
| **D_5f** | -1.08 | -6.47 | -5.01 |
| **D_9** | -1.66 | -6.00 | -4.75 |
| **D_21** | -1.35 | -5.91 | -4.93 |
| **D_23** | -1.39 | -6.28 | -4.71 |
| **D_29** | -1.05 | -7.11 | -5.55 |
| **D_32** | -0.15 | -6.82 | -6.73 |
| **D_33** | -0.72 | -6.56 | -5.67 |
| **D_40** | -0.43 | -6.56 | -6.03 |
| **D_47** | -0.68 | -7.85 | -5.76 |
| **D_49** | -0.11 | -7.51 | -6.58 |
| **GRL0617** | 0.22 | -6.66 | -6.49 |

^a^ Glide scoring energies for docking in 3E9S or 4OW0 are reported according to the similarity of compounds to co-crystallized compounds (GRL0617 in 3E9S, and C_3k in 4OW0), determined by higher %Ref and %Mol values in Table S3. Glide scoring energies for cross-docking reflect the interactions between compounds and the conformations used as receptors for cross-docking (pairs of PLpro conformations extracted from GaMD and each compound are defined in Table 2).

**Table S3**. Information corresponding to the molecular docking process and quality analysis of the poses for each of the 67 compounds in the different structures of the receptor.^a^

| **Compounds** | **LigRMSD 3E9S** | **%Ref** | **%Mol** |  | **Compounds** | **LigRMSD 4OW0** | **%Ref** | **%Mol** |
| --- | --- | --- | --- | --- | --- | --- | --- | --- |
| 6577871 | 1.22 | 47.83 | 37.93 |  | 6577871 | 0.47 | 93.10 | 93.10 |
| 7724772(R) | 1.39 | 95.65 | 100.00 |  | 7724772(R) | 1.88 | 41.38 | 54.55 |
| 7724772(S) | 1.37 | 95.65 | 100.00 |  | 7724772(S) | 1.70 | 41.38 | 54.55 |
| A_3 | 1.43 | 91.30 | 95.45 |  | A_3 | 1.93 | 41.38 | 54.55 |
| A_4 | 1.09 | 95.65 | 95.65 |  | A_4 | 2.19 | 41.38 | 52.17 |
| A_5 | 0.24 | 95.65 | 100.00 |  | A_5 | 0.31 | 41.38 | 54.55 |
| A_6 | 2.39 | 100.00 | 88.46 |  | A_6 | 0.29 | 41.38 | 46.15 |
| A_7 | 0.25 | 100.00 | 92.00 |  | A_7 | 0.19 | 41.38 | 48.00 |
| B_7a | 0.85 | 47.83 | 37.93 |  | B_7a | 0.26 | 93.10 | 93.10 |
| B_7b | 1.01 | 47.83 | 37.93 |  | B_7b | 1.39 | 93.10 | 93.10 |
| B_15a | 3.88 | 52.17 | 40.00 |  | B_15a | 0.21 | 96.55 | 93.33 |
| B_15b | 1.14 | 52.17 | 40.00 |  | B_15b | 0.18 | 96.55 | 93.33 |
| B_15c | 4.67 | 52.17 | 40.00 |  | B_15c | 0.51 | 96.55 | 93.33 |
| B_15d | 2.08 | 52.17 | 40.00 |  | B_15d | 1.29 | 96.55 | 93.33 |
| B_15e | 3.68 | 52.17 | 40.00 |  | B_15e | 1.80 | 96.55 | 93.33 |
| B_15f | 2.07 | 52.17 | 40.00 |  | B_15f | 1.14 | 96.55 | 93.33 |
| B_15g | 4.55 | 52.17 | 38.71 |  | B_15g | 0.82 | 96.55 | 90.32 |
| B_15h | 3.23 | 52.17 | 38.71 |  | B_15h | 0.62 | 96.55 | 90.32 |
| B_15i | 1.19 | 47.83 | 36.67 |  | B_15i | 0.56 | 93.10 | 90.00 |
| B_15j | 7.97 | 47.83 | 36.67 |  | B_15j | 4.04 | 93.10 | 90.00 |
| B_15k | 1.34 | 52.17 | 37.50 |  | B_15k | 1.71 | 96.55 | 87.50 |
| C_1a(R) | 0.74 | 52.17 | 37.50 |  | C_1a(R) | 1.84 | 96.55 | 87.50 |
| C_1a(S) | 3.23 | 52.17 | 37.50 |  | C_1a(S) | 2.91 | 96.55 | 87.50 |
| C_1b(R) | 2.97 | 52.17 | 37.50 |  | C_1b(R) | 2.83 | 96.55 | 87.50 |
| C_1b(S) | 4.37 | 52.17 | 37.50 |  | C_1b(S) | 0.59 | 96.55 | 87.50 |
| C_1c(R) | 3.16 | 52.17 | 36.36 |  | C_1c(R) | 2.72 | 96.55 | 84.85 |
| C_1c(S) | 5.05 | 52.17 | 36.36 |  | C_1c(S) | 1.88 | 96.55 | 84.85 |
| C_1d(R) | 2.38 | 52.17 | 32.43 |  | C_1d(R) | 2.45 | 96.55 | 75.68 |
| C_1d(S) | 4.81 | 52.17 | 32.43 |  | C_1d(S) | 3.46 | 96.55 | 75.68 |
| C_2a | 0.56 | 52.17 | 42.86 |  | C_2a | 0.17 | 96.55 | 100.00 |
| C_2b | 0.75 | 52.17 | 41.38 |  | C_2b | 0.44 | 96.55 | 96.55 |
| C_2c | 0.44 | 52.17 | 41.38 |  | C_2c | 0.36 | 96.55 | 96.55 |
| C_2d | 1.37 | 52.17 | 38.71 |  | C_2d | 2.18 | 96.55 | 90.32 |
| C_2e | 1.16 | 52.17 | 38.71 |  | C_2e | 1.93 | 96.55 | 90.32 |
| C_3a | 4.61 | 52.17 | 40.00 |  | C_3a | 0.80 | 96.55 | 93.33 |
| C_3b | 2.25 | 52.17 | 37.50 |  | C_3b | 1.63 | 96.55 | 87.50 |
| C_3c | 0.99 | 52.17 | 37.50 |  | C_3c | 1.88 | 96.55 | 87.50 |
| C_3d | 3.04 | 52.17 | 37.50 |  | C_3d | 1.49 | 96.55 | 87.50 |
| C_3e | 4.67 | 52.17 | 37.50 |  | C_3e | 0.94 | 96.55 | 87.50 |
| C_3f | 2.79 | 52.17 | 36.36 |  | C_3f | 1.61 | 96.55 | 84.85 |
| C_3g | 4.00 | 52.17 | 41.38 |  | C_3g | 0.87 | 96.55 | 96.55 |
| C_3h | 4.58 | 52.17 | 41.38 |  | C_3h | 0.61 | 96.55 | 96.55 |
| C_3i | 3.48 | 52.17 | 40.00 |  | C_3i | 2.99 | 100.00 | 96.67 |
| C_3j | 1.08 | 52.17 | 41.38 |  | C_3j | 0.30 | 96.55 | 96.55 |
| C_3k | 1.31 | 52.17 | 41.38 |  | C_3k | 1.92 | 100.00 | 100.00 |
| C_4a(R) | 7.10 | 47.83 | 37.93 |  | C_4a(R) | 2.37 | 100.00 | 100.00 |
| C_4a(S) | 7.48 | 47.83 | 37.93 |  | C_4a(S) | 3.57 | 100.00 | 100.00 |
| C_4b(R) | 8.21 | 47.83 | 37.93 |  | C_4b(R) | 0.92 | 100.00 | 100.00 |
| C_4b(S) | 7.80 | 47.83 | 37.93 |  | C_4b(S) | 1.58 | 100.00 | 100.00 |
| C_4c(R) | 7.03 | 47.83 | 37.93 |  | C_4c(R) | 2.20 | 100.00 | 100.00 |
| C_4c(S) | 8.10 | 47.83 | 37.93 |  | C_4c(S) | 1.48 | 100.00 | 100.00 |
| C_4d(R) | 8.19 | 47.83 | 37.93 |  | C_4d(R) | 2.93 | 100.00 | 100.00 |
| C_4d(S) | 8.02 | 47.83 | 37.93 |  | C_4d(S) | 0.74 | 100.00 | 100.00 |
| C_5a | 4.19 | 52.17 | 42.86 |  | C_5a | 1.58 | 96.55 | 100.00 |
| C_5b | 1.30 | 52.17 | 42.86 |  | C_5b | 2.19 | 96.55 | 100.00 |
| C_5c | 4.82 | 52.17 | 40.00 |  | C_5c | 3.20 | 100.00 | 96.67 |
| C_6a | 1.62 | 52.17 | 40.00 |  | C_6a | 2.47 | 75.86 | 73.33 |
| C_6b | 1.38 | 52.17 | 40.00 |  | C_6b | 2.47 | 75.86 | 73.33 |
| D_02 | 0.33 | 95.65 | 91.67 |  | D_02 | 0.18 | 41.38 | 50.00 |
| D_5a | 1.06 | 91.30 | 95.45 |  | D_5a | 1.45 | 41.38 | 54.55 |
| D_5b | 1.03 | 91.30 | 95.45 |  | D_5b | 1.03 | 41.38 | 54.55 |
| D_5c | 1.05 | 91.30 | 91.30 |  | D_5c | 1.31 | 41.38 | 52.17 |
| D_5d | 1.10 | 91.30 | 91.30 |  | D_5d | 1.02 | 41.38 | 52.17 |
| D_5e | 1.04 | 91.30 | 91.30 |  | D_5e | 1.04 | 41.38 | 52.17 |
| D_5f | 1.20 | 95.65 | 95.65 |  | D_5f | 1.08 | 41.38 | 52.17 |
| D_9 | 1.13 | 91.30 | 95.45 |  | D_9 | 2.47 | 41.38 | 54.55 |
| D_21 | 2.51 | 95.65 | 95.65 |  | D_21 | 0.39 | 41.38 | 52.17 |
| D_23 | 0.67 | 91.30 | 95.45 |  | D_23 | 0.21 | 41.38 | 54.55 |
| D_29 | 0.44 | 100.00 | 95.83 |  | D_29 | 1.29 | 41.38 | 50.00 |
| D_32 | 0.23 | 95.65 | 95.65 |  | D_32 | 0.17 | 41.38 | 52.17 |
| D_33 | 2.40 | 95.65 | 91.67 |  | D_33 | 0.21 | 41.38 | 50.00 |
| D_40 | 2.34 | 100.00 | 92.00 |  | D_40 | 0.48 | 41.38 | 48.00 |
| D_47 | 0.28 | 95.65 | 70.97 |  | D_47 | 0.22 | 41.38 | 38.71 |
| D_49 | 0.28 | 95.65 | 88.00 |  | D_49 | 0.18 | 41.38 | 48.00 |
| GRL0617 | 2.39 | 100.00 | 100.00 |  | GRL0617 | 0.16 | 41.38 | 52.17 |

^a^ The associated results for the structures 3E9S and 4OW0 are present at the left and the right, respectively. For LigRMSD, the references used to compare the docking poses are the compound GRL0617 co-crystallized on 3E9S, and C_3k co-crystallized on 4OW0.


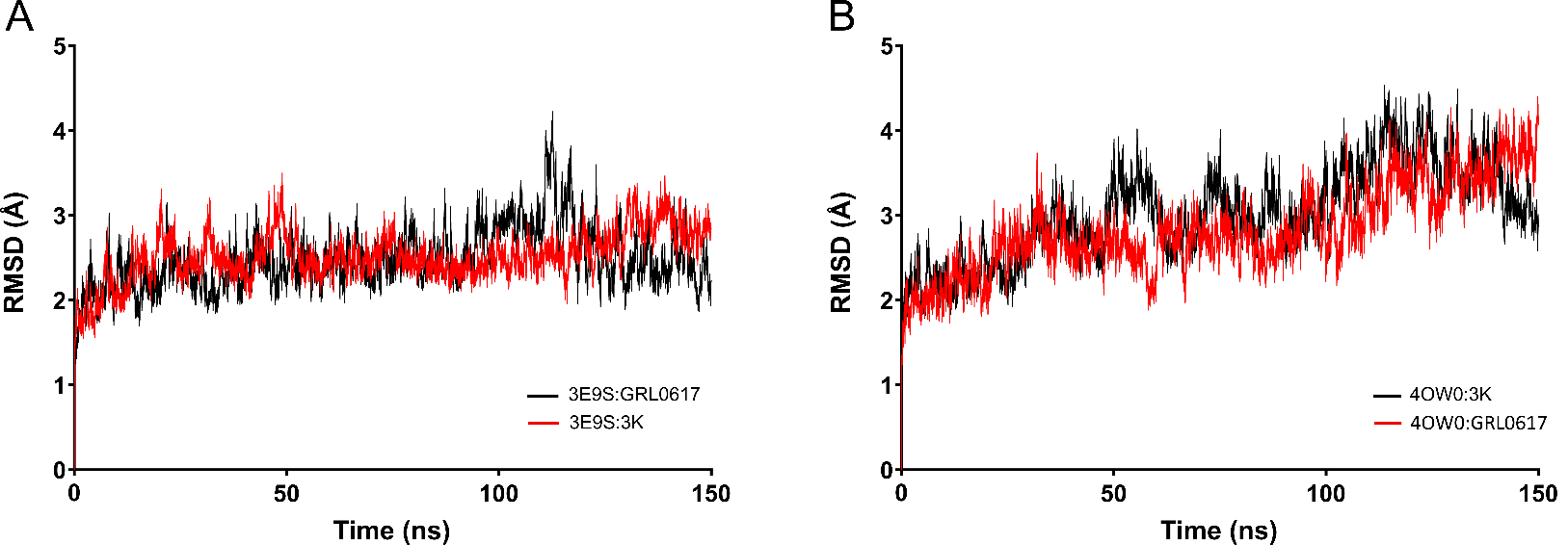


3E9S:GRL0617

3E9S: C_3k

4OW0: C_3k

4OW0: GRL0617

**Figure S2.** Time dependence of the RMSD for PLpro backbone heavy atoms extracted from GaMD simulations: **(A)** for structures with PDB code 3E9S forming complexes with GRL0617 (black line) and C_3k (red line), and **(B)** for structures with PDB code 4OW0 forming complexes with C_3k (black line) and GRL0617 (red line).


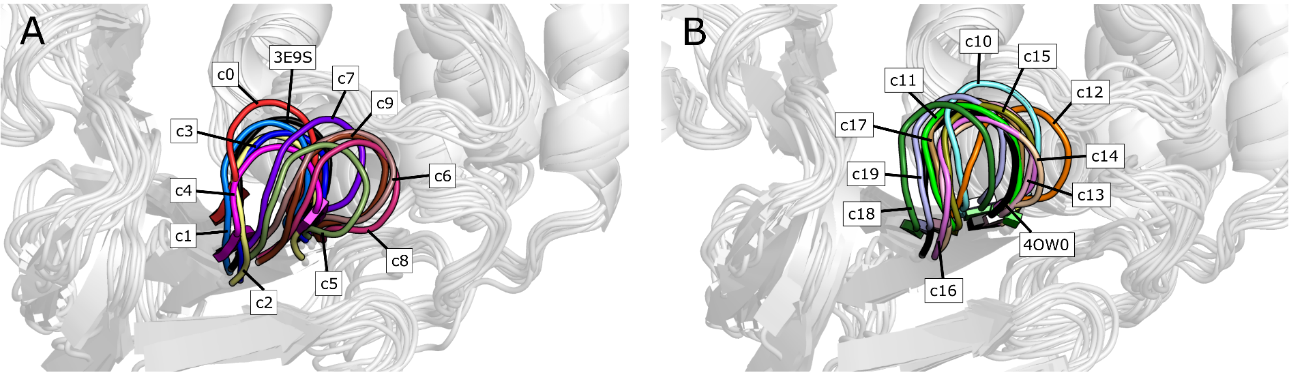


**Figure S3**. Comparison of the BL2 loop between the crystallographic structures and the 20 conformations obtained by MD and the clustering process, (A) for the structure with PDB code 3E9S and its 10 conformations (c0-c9), and (B) for the structure with PDB code 4OW0 and its 10 conformations (c10-c19).


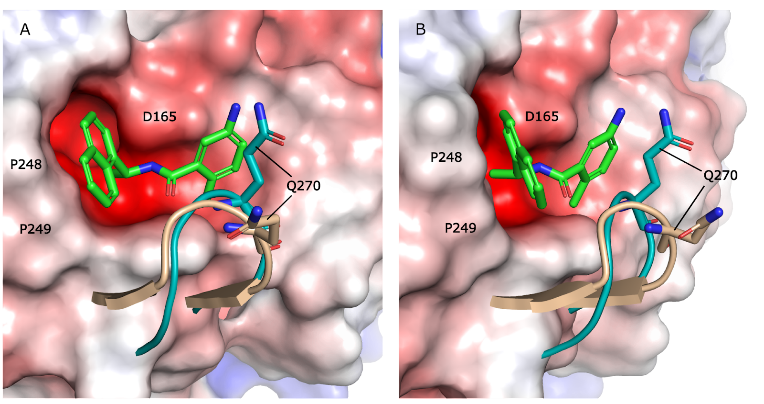


**Figure S4.** Structural difference in the BL2 blocking loop for 4OW0 and 3E9S structure of SARS-CoV-1 PLpro. A) Conformational flexibility of the BL2 loop. The loop conformation for the structure (3E9S) is depicted in blue-green, whereas for the structure 4OW0 it is marked in skin color. The PLpro structure of SARS-CoV-1 is depicted through the electrostatic surface and the Gln270 residue is used as a reference for both cases. B) 30° rotation of A.

**Table S4**. RMSD values of the binding site residues (20 residues) for the structures used in the cross-docking protocol.^a^

| - | c0 | c1 | c2 | c3 | c4 | c5 | c6 | c7 | c8 | c9 | c10 | c11 | c12 | c13 | c14 | c15 | c16 | c17 | c18 | c19 |
| --- | --- | --- | --- | --- | --- | --- | --- | --- | --- | --- | --- | --- | --- | --- | --- | --- | --- | --- | --- | --- |
| c0 | 0 |  |  |  |  |  |  |  |  |  |  |  |  |  |  |  |  |  |  |  |
| c1 | 1.57 | 0 |  |  |  |  |  |  |  |  |  |  |  |  |  |  |  |  |  |  |
| c2 | 1.82 | 1.92 | 0 |  |  |  |  |  |  |  |  |  |  |  |  |  |  |  |  |  |
| c3 | 1.88 | 1.74 | 1.95 | 0 |  |  |  |  |  |  |  |  |  |  |  |  |  |  |  |  |
| c4 | **2.09** | 1.76 | 1.84 | 1.36 | 0 |  |  |  |  |  |  |  |  |  |  |  |  |  |  |  |
| c5 | **2.67** | **2.66** | **2.42** | **2.31** | **2.44** | 0 |  |  |  |  |  |  |  |  |  |  |  |  |  |  |
| c6 | **3.19** | **3.21** | **3.20** | **2.71** | **3.12** | 1.88 | 0 |  |  |  |  |  |  |  |  |  |  |  |  |  |
| c7 | **2.32** | **2.50** | **2.39** | **2.33** | **2.67** | 1.82 | 1.81 | 0 |  |  |  |  |  |  |  |  |  |  |  |  |
| c8 | **3.02** | **3.06** | **2.87** | **2.69** | **2.96** | 1.73 | 1.53 | 1.83 | 0 |  |  |  |  |  |  |  |  |  |  |  |
| c9 | **2.69** | **2.69** | **2.57** | **2.47** | **2.66** | 1.53 | 1.73 | 1.74 | 1.13 | 0 |  |  |  |  |  |  |  |  |  |  |
| c10 | **2.32** | **2.42** | **2.83** | **2.01** | **2.38** | **2.61** | **2.42** | **2.35** | **2.69** | **2.53** | 0 |  |  |  |  |  |  |  |  |  |
| c11 | **2.39** | **2.03** | **2.37** | 1.91 | 1.79 | **2.97** | **3.42** | **2.81** | **3.34** | **2.94** | **2.05** | 0 |  |  |  |  |  |  |  |  |
| c12 | **2.82** | **2.83** | **2.83** | **2.68** | **2.96** | **2.10** | **2.06** | 1.86 | **2.19** | 1.98 | **2.34** | **2.72** | 0 |  |  |  |  |  |  |  |
| c13 | **2.09** | **2.02** | **2.18** | **2.09** | **2.12** | **2.52** | **3.18** | **2.58** | **2.91** | **2.47** | **2.49** | **2.01** | **2.50** | 0 |  |  |  |  |  |  |
| c14 | **2.47** | **2.23** | **2.28** | **2.24** | **2.15** | **2.33** | **3.05** | **2.62** | **2.77** | **2.35** | **2.62** | **2.16** | **2.28** | 1.25 | 0 |  |  |  |  |  |
| c15 | **2.23** | **2.17** | **2.40** | **2.08** | **2.27** | **2.35** | **2.88** | **2.30** | **2.80** | **2.30** | **2.27** | 1.76 | **2.08** | 1.62 | 1.68 | 0 |  |  |  |  |
| c16 | **2.12** | 1.93 | 1.90 | **2.07** | 1.97 | **2.23** | **2.99** | **2.31** | **2.77** | **2.28** | **2.37** | 1.71 | **2.10** | 1.52 | 1.46 | 1.21 | 0 |  |  |  |
| c17 | 1.94 | 1.89 | **2.33** | **2.31** | **2.29** | **2.91** | **3.40** | **2.78** | **3.24** | **2.76** | **2.35** | 1.67 | **2.68** | 1.96 | **2.07** | 1.91 | 1.59 | 0 |  |  |
| c18 | **2.15** | **2.15** | **2.31** | **2.79** | **2.54** | **3.24** | **4.07** | **3.09** | **3.93** | **3.41** | **3.16** | **2.08** | **3.07** | **2.31** | **2.50** | **2.20** | 1.84 | 1.98 | 0 |  |
| c19 | 1.94 | 1.97 | **2.18** | **2.53** | **2.44** | **3.09** | **3.79** | **2.88** | **3.65** | **3.18** | **2.82** | 1.88 | **2.73** | 1.94 | **2.12** | 1.88 | 1.49 | 1.56 | 1.29 | 0 |

^a^ RMSD values higher than 2.0 Å are in bold letter.

**Figure S5.** RMSF plot of the residues that constitute the SARS-CoV PLpro binding site in c0-c19.

**Table S5**. RMSD values of the Y269 and Q270 residues for the structures used in the cross-docking protocol.^a^

| - | c0 | c1 | c2 | c3 | c4 | c5 | c6 | c7 | c8 | c9 | c10 | c11 | c12 | c13 | c14 | c15 | c16 | c17 | c18 | c19 |
| --- | --- | --- | --- | --- | --- | --- | --- | --- | --- | --- | --- | --- | --- | --- | --- | --- | --- | --- | --- | --- |
| c0 | 0 |  |  |  |  |  |  |  |  |  |  |  |  |  |  |  |  |  |  |  |
| c1 | 1.60 | 0 |  |  |  |  |  |  |  |  |  |  |  |  |  |  |  |  |  |  |
| c2 | **2.15** | 0.97 | 0 |  |  |  |  |  |  |  |  |  |  |  |  |  |  |  |  |  |
| c3 | 1.99 | 1.32 | 0.94 | 0 |  |  |  |  |  |  |  |  |  |  |  |  |  |  |  |  |
| c4 | **3.00** | 1.75 | 1.27 | 1.43 | 0 |  |  |  |  |  |  |  |  |  |  |  |  |  |  |  |
| c5 | **4.70** | **4.49** | **3.87** | **3.20** | **3.61** | 0 |  |  |  |  |  |  |  |  |  |  |  |  |  |  |
| c6 | **5.65** | **5.79** | **5.30** | **4.51** | **5.28** | 1.86 | 0 |  |  |  |  |  |  |  |  |  |  |  |  |  |
| c7 | **3.56** | **3.83** | **3.44** | **2.66** | **3.60** | 1.74 | **2.22** | 0 |  |  |  |  |  |  |  |  |  |  |  |  |
| c8 | **6.12** | **6.14** | **5.55** | **4.86** | **5.49** | **2.06** | 0.75 | **2.67** | 0 |  |  |  |  |  |  |  |  |  |  |  |
| c9 | **5.13** | **5.17** | **4.64** | **3.91** | **4.62** | 1.45 | 0.81 | 1.69 | 1.03 | 0 |  |  |  |  |  |  |  |  |  |  |
| c10 | 1.54 | **2.48** | **2.72** | **2.09** | **3.25** | **3.81** | **4.53** | **2.49** | **5.06** | **4.08** | 0 |  |  |  |  |  |  |  |  |  |
| c11 | 1.94 | 1.49 | **2.04** | 1.86 | **2.17** | **4.48** | **5.73** | **3.84** | **6.18** | **5.22** | **2.18** | 0 |  |  |  |  |  |  |  |  |
| c12 | **4.40** | **4.60** | **4.30** | **3.45** | **4.38** | **2.15** | 1.92 | 1.80 | **2.52** | 1.84 | **3.20** | **4.29** | 0 |  |  |  |  |  |  |  |
| c13 | **2.36** | **2.33** | **2.40** | 1.67 | **2.38** | **3.19** | **4.31** | **2.56** | **4.81** | **3.87** | 1.61 | 1.54 | **2.89** | 0 |  |  |  |  |  |  |
| c14 | **2.94** | **2.57** | **2.40** | 1.66 | **2.25** | **2.74** | **3.95** | **2.52** | **4.39** | **3.51** | **2.29** | **2.02** | **2.53** | 0.94 | 0 |  |  |  |  |  |
| c15 | **2.50** | **2.63** | **2.73** | **2.09** | **2.63** | **3.35** | **4.47** | **2.64** | **4.97** | **4.03** | 1.63 | 1.74 | **3.18** | 0.76 | 1.55 | 0 |  |  |  |  |
| c16 | **2.68** | 1.97 | 1.77 | 1.33 | 1.42 | **3.16** | **4.62** | **2.93** | **4.98** | **4.07** | **2.37** | 1.52 | **3.43** | 1.19 | 1.15 | 1.45 | 0 |  |  |  |
| c17 | 1.51 | 1.53 | **2.00** | 1.58 | **2.37** | **4.11** | **5.21** | **3.27** | **5.69** | **4.71** | 1.46 | 0.79 | **3.74** | 1.19 | 1.78 | 1.46 | 1.52 | 0 |  |  |
| c18 | **2.30** | 1.82 | **2.54** | **2.67** | **2.77** | **5.49** | **6.78** | **4.89** | **7.23** | **6.28** | **3.03** | 1.17 | **5.30** | **2.61** | **3.01** | **2.80** | **2.51** | 1.74 | 0 |  |
| c19 | 1.69 | 1.65 | **2.42** | **2.34** | **2.80** | **5.10** | **6.24** | **4.29** | **6.73** | **5.74** | **2.25** | 0.82 | **4.74** | **2.11** | **2.67** | **2.23** | **2.26** | 1.06 | 0.92 | 0 |

^a^ RMSD values higher than 2.0 Å are in bold letter.


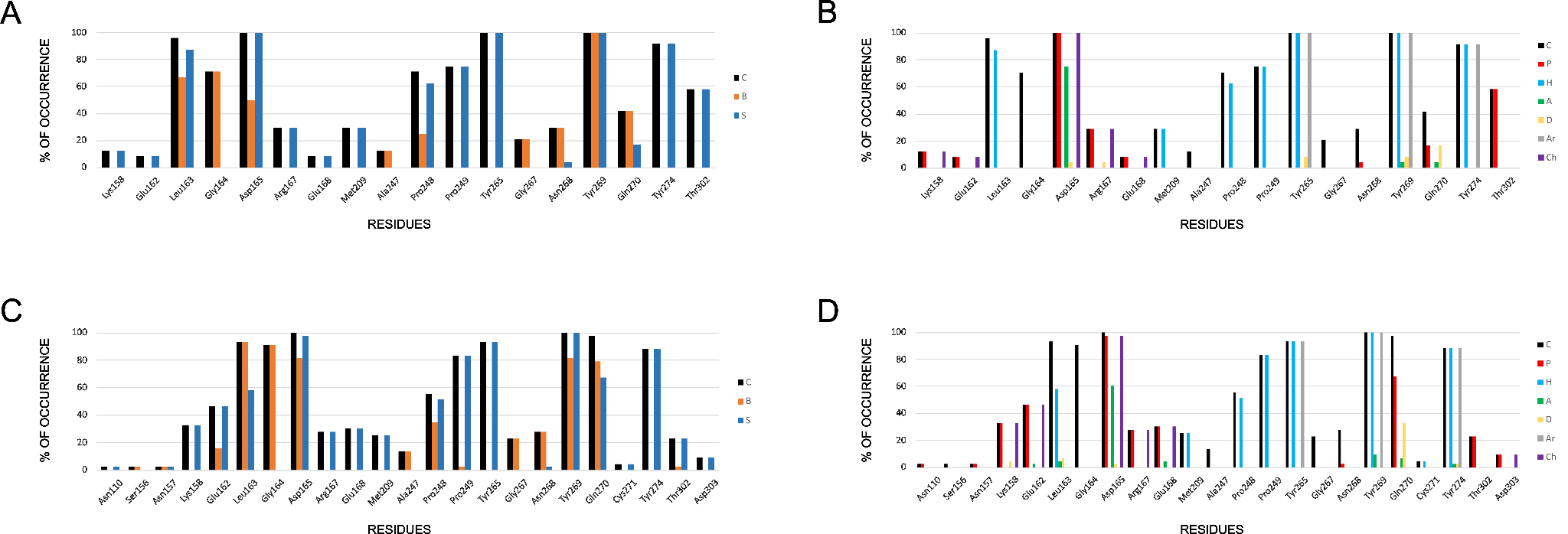


**Figure S6.** IFPs that describe interactions between the selected complexes between the cross-docked compounds and SARS-CoV-1 PLpro conformations showing the highest correlation. (A,B) Interactions of compounds from series A, D, 7724772, and GRL0617. (C,D) Interactions of compounds from series B, C, and 6577871. Interactions in the graphs at the left (A and C) are presented as percentage of occurrence of contacts [C], interactions with the backbone of the residue [B], and interactions with the side chain of the residue [S]. Interactions in the graphs at the right (B and D) are presented as percentage of occurrence of chemical interactions: contacts [C], polar [P], hydrophobic [H], HBs where the residue is an acceptor [A], HBs where the residue is a donor [D], aromatic [Ar], and electrostatic with charged groups [Ch].
